# Intraoperative tranexamic acid is associated with postoperative stroke in patients undergoing cardiac surgery

**DOI:** 10.1371/journal.pone.0177011

**Published:** 2017-05-26

**Authors:** Zhen-feng Zhou, Feng-jiang Zhang, Yang- fan Huo, Yun-xian Yu, Li-na Yu, Kai Sun, Li-hong Sun, Xiu-fang Xing, Min Yan

**Affiliations:** 1 Department of Anesthesiology, The Second Affiliated Hospital, School of Medicine, Zhejiang University, Hangzhou, China; 2 Weifang Medical University, Weifang, China; 3 Department of Epidemiology and Health Statistics, School of Public Health, Zhejiang University, Hangzhou, China; 4 Jiangsu Province Key Laboratory of Anesthesiology, Xuzhou Medical University, Xuzhou, China; Thomas Jefferson University, UNITED STATES

## Abstract

**Background:**

Stroke is a devastating and potentially preventable complication of cardiac surgery. Tranexamic acid (TXA) is a commonly antifibrinolytic agent in cardiac surgeries with cardiopulmonary bypass (CPB), however, there is concern that it might increase incidence of stroke after cardiac surgery. In this retrospective study, we investigated whether TXA usage could increase postoperative stroke in cardiac surgery.

**Methods:**

A retrospective study was conducted from January 1, 2010, to December 31, 2015, in 2,016 patients undergoing cardiac surgery, 664 patients received intravenous TXA infusion and 1,352 patients did not receive any antifibrinolytic agent. Univariate and propensity-weighted multivariate regression analysis were applied for data analysis.

**Results:**

Intraoperative TXA administration was associated with postoperative stroke (1.7% vs. 0.5%; adjusted OR, 4.11; 95% CI, 1.33 to 12.71; *p* = 0.014) and coma (adjusted OR, 2.77; 95% CI, 1.06 to 7.26; *p* = 0.038) in cardiac surgery. As subtype analysis was performed, TXA administration was still associated with postoperative stroke (1.7% vs. 0.3%; adjusted OR, 5.78; 95% CI, 1.34 to 27.89; *p* = 0.018) in patients undergoing valve surgery or multi-valve surgery only, but was not associated with postoperative stroke (1.7% vs. 1.3%; adjusted OR, 5.21; 95% CI, 0.27 to 101.17; *p* = 0.276) in patients undergoing CABG surgery only. However, TXA administration was not associated with postoperative mortality (adjusted OR, 1.31; 95% CI, 0.56 to 3.71; *p* = 0.451), seizure (adjusted OR, 1.13; 95% CI, 0.42 to 3.04; *p* = 0.816), continuous renal replacement therapy (adjusted OR, 1.36; 95% CI, 0.56 to 3.28; *p* = 0.495) and resternotomy for postoperative bleeding (adjusted OR, 1.55; 95% CI, 0.55 to 4.30; *p* = 0.405). No difference was found in postoperative ventilation time (adjusted B, -1.45; SE, 2.33; *p* = 0.535), length of intensive care unit stay (adjusted B, -0.12; SE, 0.25; *p* = 0.633) and length of hospital stay (adjusted B, 0.48; SE, 0.58; *p* = 0.408).

**Conclusions:**

Based on the 5-year experience of TXA administration in cardiac surgery with CPB, we found that postoperative stroke was associated with intraoperative TXA administration in patients undergoing cardiac surgery, especially in those undergoing valve surgeries only. This study may suggest that TXA should be administrated according to clear indications after evaluating the bleeding risk in patients undergoing cardiac surgery, especially in those with high stroke risk.

## Introduction

Postoperative bleeding is a major complication in cardiac surgery [1]. Concern about potential hemorrhagic risk of cardiac surgery may influence physicians to continue antifibrinolytic agents, thus increasing stroke risk in those patients [2]. Tranexamic acid (TXA), a synthetic antifibrinolytic drug, has been increasingly used to manage patients for preventing perioperative bleeding in cardiac surgery, acting by inhibiting tissue plasminogen and plasmin. Many randomized trials have found that TXA could decrease blood loss and allogeneic transfusion in patients undergoing cardiac surgery [2, 3].

Stroke is another disaster cerebrovascular complication after cardiac surgery and is linked to a poorer outcome [4]. Some studies have shown that TXA increases the risk of stroke [5]. Furthermore, although a meta-analysis noted that either mortality or thromboembolic events was few and was not increased in TXA group compared to non-treatment group, the authors still admonish for routine use of TXA in cardiac surgery [6]. However, a recently large and randomized study found that TXA was not associated with a higher risk of cerebral infarction in patients undergoing coronary-artery (CABG) surgery [7]. Another important point was that patients of CABG surgery were always with atherosclerosis, so the overload of thrombosis and the potential cause for postoperative stroke was not completely consistent between patients undergoing CABG surgery and other type of cardiac surgery including valve surgery.

The neurological risks of TXA use including stroke and thromboembolic events which are still undetermined [8, 9]. The aim of this study was to investigate the relationship between TXA and postoperative stroke in patients undergoing cardiac surgery.

## Materials and methods

**Fig 1:**
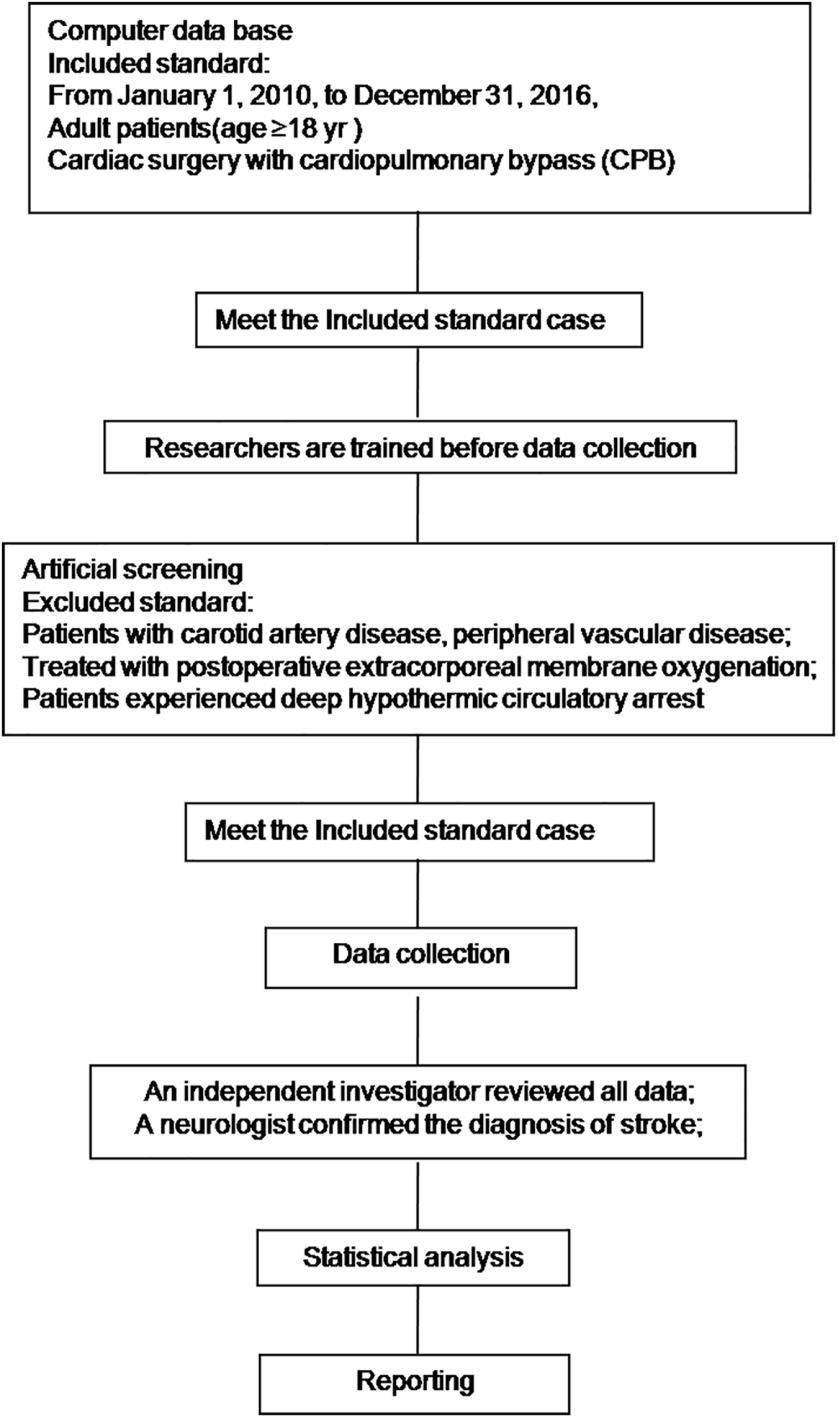
Study flowchart.

This study was a single university medical center retrospective study and an independent investigator reviewed and saved all data by the Excel form (Fig 1). All records relating to the subject's identity were kept confidential and were not disclosed according to the laws. Data was collected and was reported to the data processing center anonymously. The laboratory data was stored in a separate, safe place and it was accessible only by the relevant laboratory personnel. Any published data, subjects and investigators were subjected to data protection laws. So the need for written informed consent from the participants was waived by The Ethics Committee of Second Affiliated Hospital of Zhejiang University (Hangzhou, China; No.2016-021). From January 1, 2010, to December 31, 2015, 2,175 consecutive adult patients underwent cardiac surgery with cardiopulmonary bypass (CPB). All individual participants were discharged before study was started. Patients with carotid artery disease, peripheral vascular disease, experienced deep hypothermic circulatory arrest or treated with postoperative extracorporeal membrane oxygenation were excluded.

General anesthesia and standard monitoring were performed. CPB components and techniques did not change during the study period. According to institutional standards, body temperature of patients was maintained at 30°C during CPB. When the surgery was finished, patients were rewarmed to 37°C and weaned from CPB. The cell saver was routinely performed. Intraoperative TXA was the only antifibrinolytic agent used, and this was administered throughout all study period according to our institution’s protocol of 1 g before sternotomy, followed by a continuous infusion of 400 mg/h throughout the operation, with added 500 mg to the extracorporeal circuit. The administration of intraoperative TXA was according to the anesthesiologist’s decision and it was identified by the anesthesiologist and pharmacist based on anesthesia and pharmacy dispensing records. An additional 1 g of TXA was given according to the surgeon’s decision. Point-of-care testing, such as thrombelastography (TEG), allowed rapid identification of the activation of the fibrinolytic system and was performed according to the anesthetist’s decision. The trans-esophageal echocardiography (TEE) technique was used for hemodynamic and cardiac function monitoring, and for monitoring air bubble and particulate embolic material. Blood transfusions were usually carried out when the following criteria were met [2] and additional decisions were made based on the patient’s clinical condition. Red blood cells (RBCs) were transfused when hemoglobin concentration was <6 g/dL during CPB and <7 g/dL after CPB. When international normalized ratio (INR) > 1.4 or activated partial prothrombin time (aPTT)> 50 sec or R time > 10 min in Thromboelastometry test (TEG), prothrombin complex concentrate (PCC) was administered (20–30 IU/kg). If the above mentioned parameters prolongation did not respond to administration of PCC or there occured hypovolemia, Fresh frozen plasma (FFP) was transfused (10–15 mL/kg). Platelets were transfused when platelets count < 50 × 109/L and fibrinogen concentrate (Fb) was administered of 25–50 mg/kg when Fb < 1.5 g/L. Activated recombinant factor VII (rVIIa) was considered of 90 ug/kg as rescue therapy if the bleeding still existed while INR < 1.4, aPTT< 50 sec, Fb > 2 g/L, platelet count > 100 × 109/L. The withdrawn whole blood was reinfused first when transfusion criteria were met or reinfused at the end of operation even if transfusion criteria were not met in the Acute Normovolemic Hemodilution (ANH) group. The withdrawn whole blood was reinfused within 6 h. Blood recovered from the extracorporeal circuit system (pumped blood) was not accounted into the volume of the blood transfusions.

Perioperative variables including demographic characteristic, individual history, preexisting risk factors, preoperative medications, co-morbidities, intraoperative data, stroke and other postoperative complications were retrieved from the hospital medical records and were organized according to the STS National Adult Cardiac Surgery Database criteria, which occurred during the hospitalization, including the entire postoperative period up to discharge even if > 30 days. Laboratory test and part of preoperative characteristics were automated deriving from computer data base. Researchers were trained before data collection and an independent investigator reviewed all data. The researchers collecting postoperative end points were blinded to preoperative and intraoperative characteristics. On the basis of the STS criteria and previous literature, the following definitions were used.

*Postoperative Stroke Diagnosis.* Postoperative stroke was identified as a new-onset of neurological deficit symptoms secondary to an ischemic cerebrovascular accident, which was confirmed by radiological data (computed tomography or magnetic resonance imaging), and persisted for more than 24 h [10]. A neurologist confirmed the diagnosis by reviewing the hospital records in all cases.

*Other Postoperative End Points.* Other end points included in-hospital mortality and morbidities, duration of intensive care unit (ICU) stay, length of hospital stay (LOS) and duration of mechanical ventilation. Hospital morbidities included the following parts: (1) The definition of seizure was a new-onset neuropsychiatric disorder with increased motor activity of agitated or hyperactive state [11]. (2) Coma was defined as a new postoperative coma that lasted for more than 24 h following thromboembolic event, cerebral hemorrhage or anoxic/ischemic encephalopathy. (3) The requirement of postoperative in-hospital continuous renal replacement therapy (CRRT) [12]. (4) Resternotomy was performed for bleeding during hospitalization.

### Statistical methods

The student’s *t*-test was used to test for normal continuous variables, Mann–Whitney *U*-test was used for non-normality continuous variables, and the Chi-squared or Fisher’s exact tests were used for categorical data. Missing continuous variables of baseline parameters were less than 10% and were replaced by median in Tables 1 and 2.

**Table 1:** Demographic and clinical characteristics.

Preoperative Characteristics	TXA		*p*-value
	Yes (N = 664)	No (N = 1352)	
Age [mean(SD); yr]	51 ± 13	53 ± 13	0.019
Male/female, no. (%)	296/368(44.6%)	661/691(48.9%)	0.068
BMI [mean(SD); kg/m^2^]	22.0 ± 3.0	22.4 ± 3.1	0.002
ASA, no. (%)			0.005
I	5(0.8%)	1(0.1%)	
II	136(20.5%)	261(19.3%)	
III	458(69.0%)	994(73.5%)	
IV	62(9.3%)	95(7.0%)	
V	3(0.5%)	1(0.1%)	
NYHA class III/IV, no.(%)	230(34.6%)	351(26.0%)	<0.001
History of smoking, no. (%)	142(21.4%)	342(25.3%)	0.053
**Coexistent disease**			
AF, no. (%)	231(34.8%)	255(18.9%)	<0.001
Hypertension, no. (%)	171(25.8%)	298(22.0%)	0.064
Diabetes, no. (%)	41(6.2%)	108(8.0%)	0.144
HLP, no. (%)	1(0.2%)	15(1.1%)	0.029#
Cerebrovascular disease, no. (%)	40(6.0%)	77(5.7%)	0.767
CKD, no. (%)	12(1.8%)	25(1.8%)	0.947
Liver dysfunction, no. (%)	4(0.6%)	16(1.2%)	0.338#
COPD, no. (%)	10(1.5%)	23(1.7%)	0.746
Infective Endocarditis, no. (%)	13(2.0%)	27(2.0%)	0.953
MI, no. (%)	14(2.1%)	47(3.5%)	0.092
Preoperative shock, no. (%)	0(0%)	2(0.1%)	1#
Anemia, no. (%)	80(12.1%)	197(15.0%)	0.088
**Preoperative Medication**			
ARB or ACEI, no. (%)	58(8.7%)	101(7.5%)	0.322
β-blockers, no.(%)	41(6.2%)	122(9.0%)	0.027
Calcium Channel Blockers, no. (%)	58(8.7%)	70(5.2%)	0.002
Nitrates, no. (%)	19(2.9%)	68(5.0%)	0.024
Coumadin, no. (%)	12(1.8%)	73(5.4%)	<0.001
Heparin, no. (%)	0(0%)	4(0.3%)	0.309#
Clopidogrel, no. (%)	18(2.7%)	61(4.5%)	0.050
Aspirin, no. (%)	48(7.2%)	147(10.9%)	0.009
Statin use, no.(%)	24(3.6%)	76(5.6%)	0.051
Diuretics, no. (%)	43(6.5%)	125(9.2%)	0.034
Digoxin, no. (%)	29(4.4%)	75(5.5%)	0.260
**Preoperative laboratory examination**			
LVEF <35%, no.(%)	5(0.8%)	9(0.7%)	0.824
T-ch [mean(SD); mmol/L]	4.3 ± 1.0	4.3 ± 1.1	0.153
BUN [mean(SD); mmol/L]	5.9 ± 2.3	6.0 ± 2.3	0.408
Hb [mean(SD); g ·L^-1^]	132 ± 19	131 ± 18	0.116
PLt [mean(SD); 10^3^/mm^3^]	169 ± 58	171 ± 58	0.378
INR [mean(SD); seconds]	1.07 ± 0.24	1.09 ± 0.28	0.154
Propensity score [median(SD)]	0.50 ±0.22	0.25 ±0.19	< 0.001

#: Fisher’s exact test was used

BMI = body mass index; ASA = American Society of Anesthesiologists; NYHA = New York Heart Association; AF = atrial fibrillation; HLP = Hyperlipidaemia; CKD = chronic kidney disease; COPD = chronic obstructive pulmonary disease; MI = myocardial infarction in 30 days before operation; ARB = angiotensin receptor blockers; ACEI = angiotensin converting enzyme inhibitors; LVEF = left ventricular ejection fraction; T-ch = serum cholesterol; BUN = serum urea nitrogen; Hb = hemoglobin; PLt = platelet count; INR = international normalized ratio; TXA = Tranexamic acid.

**Table 2:** Operative characteristics

Characteristics	TXA		*p*-value
	Yes (N = 664)	No (N = 1352)	
Redo surgery	14(2.1%)	15(1.1%)	0.077
Emergent operation	3(0.5%)	17(1.3%)	0.098#
**Type of surgery, no. (%)**			0.058
CABG Only	58(8.7%)	156(11.5%)	
Aortic valve	90(13.6%)	151(11.2%)	
Mitral valve	124(18.7%)	246(18.2%)	
Tricuspid valve	37(5.6%)	61(4.5%)	
Complex cardiac	255(38.4%)	486(35.9%)	
Transplant	2(0.3%)	4(0.3%)	
Aortic	18(2.7%)	69(5.1%)	
Others	80(12.0%)	179(13.2%)	
Duration of anesthesia [mean(SD); min]	315 ± 99	309 ± 113	0.239
Duration of surgery [mean(SD); min]	266 ± 94	265 ± 109	0.770
CPB time [mean(SD); min]	131 ± 63	128 ± 63	0.370
IABA, no. (%)	3(0.5%)	7(0.5%)	1#
**Input and Output Characteristics**			
Introperative Crystals [mean(SD); mL]	1226 ± 451	1657 ± 582	<0.001
Introperative Colloid [mean(SD); mL]	770 ± 405	648 ± 357	<0.001
Cell salvage transfusion[median(IQR); mL]	400(250~526)	300(200~500)	<0.001
ANH [median(IQR); mL]	0(0~0)	0(0~0)	0.439
Pump blood [mean(SD); mL]	501 ± 95	524 ± 150	<0.001
Blood loss [mean(SD); mL]	789 ± 450	625 ± 377	<0.001
Urine output [mean(SD); mL]	793 ± 453	755 ± 511	0.087
**Intra-operative transfusion**			
RBCs transfusion, no. (%)	155(23.3%)	312(23.1%)	0.894
FFP transfusion, no. (%)	274(41.3%)	468(34.6%)	0.004
Platelet transfusion, no. (%)	82(12.3%)	99(7.3%)	<0.001
**Intraoperative coagulation drugs**			
PCC, no. (%)	28(4.2%)	30(2.2%)	0.012
Fb, no. (%)	31(4.7%)	35(2.6%)	0.014
rFVIIa, no. (%)	2(0.3%)	6(0.4%)	1#
**Laboratory Characteristics during the first post-operative morning**			
Hct [mean(SD); %]	30 ± 5	30 ± 5	0.195
PLt [mean(SD); 10^3^/mm^3^]	110 ± 45	112 ± 43	0.324
INR [mean(SD); seconds]	1.38 ± 0.21	1.35 ± 0.26	0.065

#: Fisher’s exact test was used

Redo surgery = ≥1 previous sternotomy; CABG = coronary artery bypass grafting; Aortic valve surgery = without ascending aortic replacement; Complex cardiac surgery = combined coronary artery bypass graft surgery and valve surgery or multi-valve surgery; Aortic surgery = Aortic dissections, type A and B, thoracic aortic aneurysms) or Aortic valve surgery with ascending aortic replacement; Others surgery type including ASD (atrial septal defect), VSD (interventricular septal defect), LAM (atrial myxoma), ASV (Aneurysm Sinus Valsalva), CPF (coronary artery pulmonary artery fistula), patent foramen ovale (PFO)/atrial septal aneurysm surgery, and surgery for cardiac tumors; CPB = cardiopulmonary bypass; IABA = intra-aortic balloon pump; ANH = Acute Normovolemic Hemodilution; RBCs = red blood cells; PCC = Prothrombin Complex Concentrate; Fb = Fibrinogen concentrate; rFVIIa = recombinant activated factor VII; Hct = hematocrit; PLt = platelet count; INR = international normalized ratio; TXA = Tranexamic acid.

We calculated the propensity score to reduce selection bias in patients who received TXA. A propensity score with a calliper width of 0.1 of the conditional probability of each patient receiving TXA was generated from a multivariable logistic regression model on the basis of the covariates using treatment type (received TXA and not) as a binary dependent variable, with patient demographic and clinical risk factors (Table 1) as independent variables. The variables in the propensity model were listed as following: age, gender, body mass index (BMI), ASA, NYHA, History of smoking, atrial fibrillation (AF), Hypertension, Hyperlipidaemia, myocardial infarction in 30 days before operation (MI), Anemia, β-blockers, Calcium Channel Blockers, Nitrates, Coumadin, Clopidogrel, Aspirin, Statin use, Diuretics and type of surgery.

The propensity-weighted multivariate regression analyses were used to reduce distortion in which we used the estimated propensity score as weights for patients who received TXA and also added TXA administration as an independent factor to the model. All multivariate regression analyses assessing postoperative stroke and other end points included the basic characteristics as follows: TXA administration, propensity score, age, gender and New York Heart Association (NYHA) class. Additional risk factors as listed in Tables 1 and 2 were chosen deliberately according to previous literature and those variables with statistical differences of *p* ≤ 0.1 in the univariate analysis. The Poisson regression analysis was applied to assess stroke and seizure, coma, mortality, CRRT and resternotomy for postoperative bleeding, and linear regression analysis with the enter selection procedure was used to assess ventilation time, ICU and LOS stay including the same additional risk factors of postoperative mortality. The poisson regression analysis assessing stroke and seizure included additional risk factors as following: history of smoke and cerebrovascular disease, serum level of platelet count and cholesterol, preoperative medication of clopidogrel and aspirin, duration of anesthesia and surgery, administration of colloid (A and B in S1 Table).

Results are reported as odds ratios (OR) with 95% confidence intervals (CI) or beta (B) with standard error (SE). *p* <0.05 was considered to be statistically significant and all reported *p* values were 2-sided. Statistical analysis was performed using SAS version 8.0 for Windows (SAS Inc, Cary, NC).

## Results

### Baseline parameters and operative characteristics

**Fig 2:**
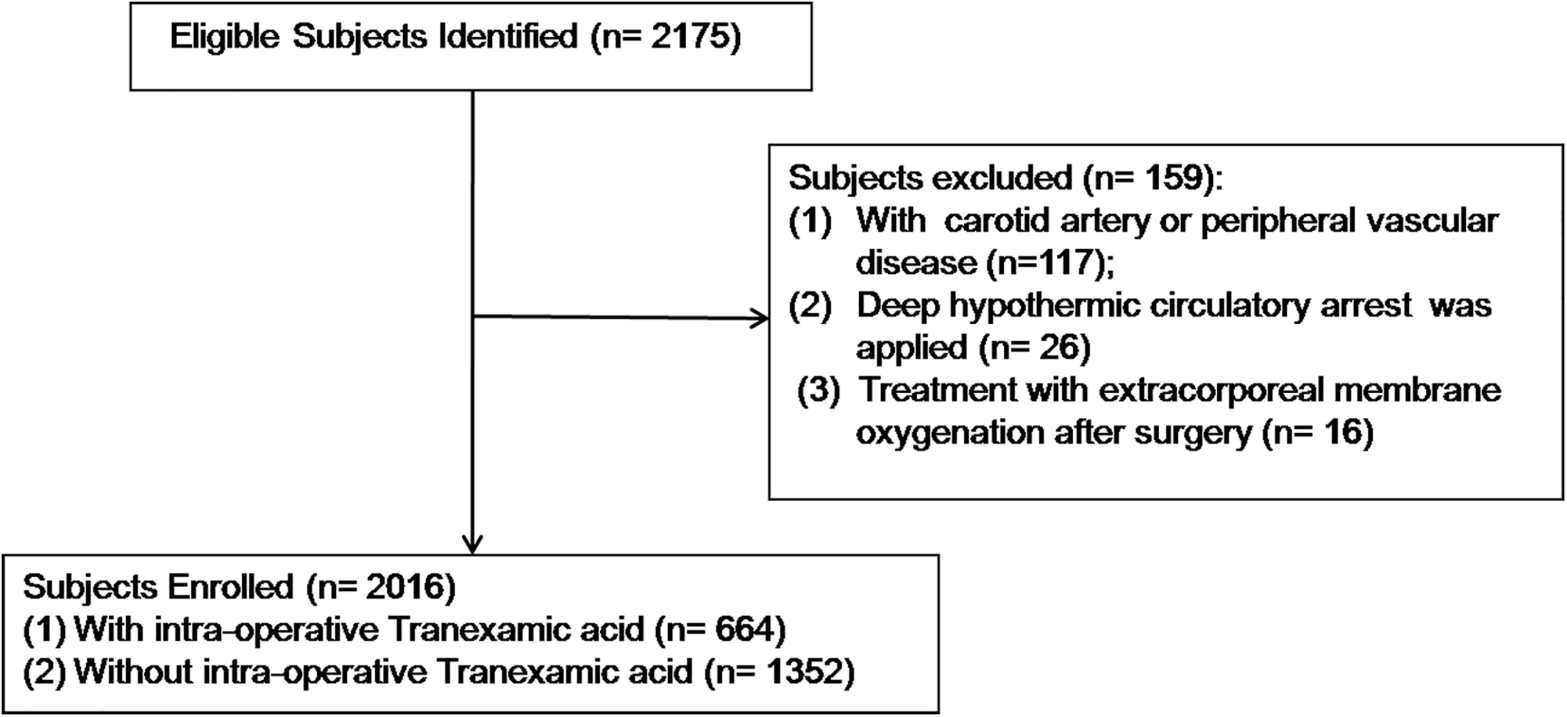
Study population recruitment summary.

A total of 2,016 patients were identified, they were divided into a TXA group (n = 664), and a non-TXA group (n = 1,352) (Fig 2). Patients in the non-TXA group did not receive any antifibrinolytic drug. The average age of the study population was 52 years, 52.5% were females, 80.0% had ASA classes III, IV, and V, and 28.8% had NYHA classes III and IV. A total of 41.1% of the total patients underwent complex cardiac (combined coronary artery bypass graft surgery and valve surgery or multi-valve surgery) or aortic surgery (Aortic dissections, type A and B, thoracic aortic aneurysms or Aortic valve surgery with ascending aortic replacement).

The basic clinical features of the two groups are presented in Table 1. Compared with the non-TXA group, the patients in the TXA group presented with more NYHA class III/IV (34.6% vs. 26.0%, *p* <0.001), younger age (51 ±13 vs. 53 ±13, *p* = 0.019), lower BMI (22.0 ± 3.0 vs. 22.4 ± 3.1, *p* = 0.002), greater ASA class (*p* = 0.005), greater incidence of previous AF (34.8% vs. 18.9%, *p* <0.001), less hyperlipidemia (0.2% vs. 1.1%, *p* = 0.029), more administration of Calcium Channel Blockers (8.7% vs. 5.2%, *p* = 0.027), less frequent administration of β-blockers (6.2% vs. 9.0%, *p* = 0.027), nitrates (2.9% vs. 5.0%, *p* = 0.024), coumadin (1.8% vs. 5.4%, *p* <0.001), aspirin (7.2% vs. 10.9%, *p* = 0.009) and diuretics (6.5% vs. 9.2%, *p* = 0.034). The propensity score was significantly different between the two groups who received TXA and not (0.50 ± 0.22 vs. 0.25 ± 0.19, *p* <0.001).

The patients in the TXA group also had evidence of diffuse bleeding (789 ± 450 vs. 625 ± 377, *p* < 0.001) with more transfusion of FFP (41.3% vs. 34.6%, p = 0.004) and PLt (12.3% vs. 7.3%, *p* <0.001), more cell salvage (400 vs. 300; p < 0.001) but less pump blood (501 ± 95 vs. 524 ± 150, p < 0.001) transfusion, more colloid (770 ± 405 vs. 648 ± 357, *p* < 0.001) input and less crystals (1226 ± 451 vs. 1657 ± 582, *p* < 0.001) input, more administration of PCC (4.2% vs. 2.2%, p = 0.012) and Fb (4.7% vs. 2.6%, p = 0.014). However, there was no statistical difference with regard to type of surgery, duration of CPB and RBCs transfusion (Table 2).

### Postoperative stroke and complications

Forty-five of the 2,016 total patients (2.2%) died in the hospital and patients who died during the operation were excluded from postoperative stroke and other postoperative end points analysis (n = 8). Only two patients showed postoperative cerebral hemorrhage. Five of the total 18 patients with postoperative stroke (27.8%), who could not be awakened after surgery, were confirmed by radiological test. Among them, eleven patients experienced postoperative stroke (61.1%) within three days after surgery (S1 Fig) Postoperative stroke was not found in any patients undergoing aortic valve surgery or heart transplantation. CABG surgery had the highest rate of postoperative stroke (1.4%), while complex surgery, accounted for 1.2%. The incidence of postoperative stroke in other surgical types was list in S2 Fig.

Eighteen of the 2,016 total patients (0.9%) experienced postoperative stroke; the occurrence of stroke was 1.7% in the TXA group and 0.5% in the non-TXA group, respectively. The details of patients with postoperative stroke were listed in A and B in S1 Table. Postoperative stroke (1.7% vs. 0.5%; adjusted OR, 4.11; 95% CI, 1.33 to 12.71; *p* = 0.014) and coma (1.8% vs. 1.0%; adjusted OR, 2.77; 95% CI, 1.06 to 7.26; *p* = 0.038) was significantly increased in patients receiving intraoperative TXA administration. As subtype analysis was performed, TXA administration was still associated with postoperative stroke (1.7% vs. 0.3%; adjusted OR, 5.78; 95% CI, 1.34 to 27.89; *p* = 0.018) in patients undergoing valve surgery or multi-valve surgery only, but was not associated with postoperative stroke (1.7% vs. 1.3%; adjusted OR, 5.21; 95% CI, 0.27 to 101.17; *p* = 0.276) in patients undergoing CABG surgery only (Table 3). Furthermore, TXA administration was associated with postoperative stroke (1.5% vs. 0.4%; adjusted OR, 4.39; 95% CI, 1.19 to 16.19; *p* = 0.026) even if excluding the patients undergoing CABG surgery, but was not with coma (0.2% vs. 0.6%; adjusted OR, 1.25; 95% CI, 0.10 to 16.23; *p* = 0.866) (A in S2 Table). However, there were no statistical differences in postoperative mortality, seizure, CRRT and resternotomy for postoperative bleeding. No difference was found in postoperative ventilation time, length of ICU stay and LOS stay (Tables 3 and 4). The details of patients with other postoperative complications were listed as following: C and D in S1 Table for seizure, E and F in S1 Table for coma, G and H S1 Table for postoperative mortality, I and J S1 Table for CRRT and K and L S1 Table for resternotomy. After excluding the patients undergoing CABG surgery, the details of patients with postoperative outcomes were listed in S3 Table.

**Table 3:** Postoperative complications.

Outcomes	Entire Sample		Adjusted OR	*p*-value
	TXA	Non-TXA		
**Stroke**				
no	652(98.3%)	1338(99.5%)	Ref	
yes	11(1.7%)	7(0.5%)	4.11 (1.33~12.71)	0.014
** In the CABG surgery only**				
Stroke				
no	57(98.3%)	154(98.7%)	Ref	
yes	1(1.7%)	2(1.3%)	5.21 (0.27~101.17)	0.276
** In the valve surgery or multi-valve surgery only**				
Stroke				
no	475(98.3%)	889(99.7%)	Ref	
yes	8(1.7%)	3(0.3%)	5.78 (1.34~24.89)	0.018
**Seizure**				
no	655(98.8%)	1329(98.8%)	Ref	
yes	8(1.2%)	16(1.2%)	1.13 (0.42~3.04)	0.816
**Coma**				
no	651(99.8%)	1332(99.4%)	Ref	
yes	12(1.8%)	13(1.0%)	2.77 (1.06~7.26)	0.038
**Death**				
no	652(98.3%)	1319(98.1%)	Ref	
yes	11(1.7%)	26(1.9%)	1.31 (0.56~3.71)	0.451
**CRRT**				
no	649(97.9%)	1323(98.4%)	Ref	
yes	14(2.1%)	22(1.6%)	1.36 (0.56~3.28)	0.495
**Resternotomy for postoperative bleeding**				
no	656(98.9%)	1326(98.6%)	Ref	
yes	7(1.1%)	19(1.4%)	1.55 (0.55~4.30)	0.405

CRRT = continuous renal replacement therapy; TXA = Tranexamic acid.

**Table 4:** Ventilation time, ICU and LOS stay.

	TXA		Adjusted B (SE)	*p-* valve
	Yes (n = 655)	No (n = 1331)		
Ventilation [median(IQR); hours]	16(10~20)	16(8~20)	-1.45 (2.33)	0.535
ICU [median(IQR); days]	5(3~6)	4(2~6)	-0.12 (0.25)	0.633
LOS [median(IQR); days]	12(9~16)	10(8~14)	0.48 (0.58)	0.408

ICU = intensive care unit; LOS = length of hospital stay; TXA = Tranexamic acid.

## Discussion

Postoperative stroke is significantly associated with mortality and may double the expenditure of hospitalization [4]. In this analysis of consecutive patients undergoing cardiac surgery in our institution, we found that intraoperative TXA administration was associated with postoperative stroke and coma after multivariate regression model statistical adjustment. However, TXA administration was not associated with postoperative stroke in patients undergoing CABG surgery only, but was still associated with postoperative stroke in patients excluding the CABG surgery or undergoing valve surgery only.

Recently, Myles et al. [7] also found that TXA did not increases the risk of stroke after CABG surgery, which included most patients who were at the lowest risk for thrombosis and almost 71.9% of patients received aspirin within 3 days preoperation and about 80.0% of patients received postoperative aspirin within 24 hr. The obvious differences between our study and the study by Myles PS et al were that the observational vs randomized study, cardiac surgery vs CABG surgery only and the different medication of TXA or aspirin. The adverse stroke effect in this study could be explained by patient selection (only 3.3% of the surgeries in our cohort were combined CABG and cardiac-valve replacement, 9.9% were Isolated CABG, and more than 38.7% were complex cardiac and aortic surgeries), the different dose regimen of TXA (load dose of 1.0 g, followed by a continuous infusion of 400 mg/h vs 100 or 50 mg/kg), and the less use of preoperation aspirin in only 9.7% of patients. Aspirin is widely used to prevent thrombosis.

Mechanisms of postoperative stroke are commonly associated with hypoperfusion [13] and embolization [14]. The internal surface of the extracorporeal circulatory pump system was foreign material to blood that creates a unique susceptibility to the formation of microemboli, even though patients were fully heparinized. CPB causes fibrinolysis first and then results in thrombosis. It was also important to point out that a hypofibrinolytic state could occur after operation and the individual variabilities in response to CPB. Paramo et al. [15] found that plasminogen activator inhibitor 1 (PAI-1) activity was increased and tissue plasminogen activator (t-PA) levels were reduced following the fibrinolytic shut down after CPB, implying a possible effect on postoperative thrombotic complications. PAI-1 secretion was increased 15-fold 2 hours after surgery and might continue into the first postoperative day [16]. This increase was associated with an increased risk of coronary graft occlusion [17]. There were approximately one-third of patients showing no tPA increase during CPB or postoperative PAI-1 increase [18]. This individual variability was one of the reasons it was difficult to predict which patients were at risk for bleeding versus thrombosis.

At the first consideration, TXA may contribute to the systemic hyperthrombotic process, which may lead to neurologic events [19]. Slaughter et al.[20] found that antifibrinolytic therapy e-aminocaproic acid reduced the fibrinolytic activity, but not reduced thrombin generation and soluble fibrin simultaneously, suggesting a possible potential hypercoagulable prothrombotic status during the postoperative period. Another point to be noted is that some patients appear to tend to form clots, which remind us of individual differences in response of CPB [21]. Another rat trial showed that TXA could not only inhibit fibrinolysis but also even strengthen thrombus weight and clot formation [22]. There were also some other reports about the occurrence of catastrophic intracardiac or intrapulmonary thromboses after TXA administration for menorrhagia, whose speculative cause was also antifibrinolytic drugs [23]. Another randomised clinical trial reported of five severe thromboembolic incident cases in TXA group for treating upper gastrointestinal bleeding in patients with liver diseases, but the difference was not significant [24]. Furthermore, a recent study occasionally found that TXA was associated with early cerebral stroke at extubation in patients undergoing cardiac surgery [25], however, its result remains unconfirmed for lacking sufficient analysis support.

Recent studies reported a total incidence of stroke at 1.6–2.0% during the 30-day postoperative period [8] and 1.5% during hospital stay [4]; we found 0.9% patients experienced in-hospital postoperative stroke. However, postoperative stroke may be underestimated. Diffusion weighted MRI identified that small cerebral infarcts occurred in 77% of patients undergoing transcatheter aortic valve implantation, but only 6% were diagnosed on clinical grounds [26]. Furthermore, slight deficits of stroke tend to remain undiagnosed during the postoperative period, as patients are often still receiving sedative medications or experiencing pain, which may obscure subtle neurologic symptoms [27].

Seizure is certainly another adverse neurocognitive outcome after cardiac surgery and its reported incidence was about 0.9% [28]. Multiple current studies have shown that high doses of TXA could increase the incidence of seizure in patients undergoing cardiac surgery, and a dose-response relationship was observed [11, 29]. We identified 1.1% patients with seizure, but they were not related to TXA. This may be due to the lower dose of TXA administration in our study and because many seizures were witnessed only by the nurses who were not trained in assessing neurological symptoms [30].

TXA has been demonstrated to decrease blood loss and allogenic blood transfusion in cardiac surgery [2], but was not associated with lower resternotomy for postoperative bleeding [31]. We also found no significant difference in benefit of TXA used for re-explorations. Even Ovrum E et al. did not recommend routine TXA a dministration because of its weak blood saving effect and its potential risk for subsiding thrombosis [32]. Another concern is that postoperative blood loss is significantly associated with alteration in platelets and increased fibrinogen degradation simultaneously, but not with one change alone [33]. Fibrinolysis was common during CPB, but it could rapidly return to baseline levels after CPB [34]. Harker et al concluded that the severity of postoperative bleeding in patients undergoing CPB was largely due to defective platelet [35]. Görlinger K et al. suggested that the available device of point-of-care coagulation testing combined with a goal-target therapy might help reduce prophylactic antifibrinolytic usage if bleeding occurred [36]. So TXA administration may need to be based on clear indications after evaluating the bleeding risks.

Eight patients (0.4%) died in the operating room and 33 patients (1.8%) died postoperatively in the hospital. No difference was found in postoperative in-hospital mortality, ventilation time and ICU stay between those who received TXA and those who did not in our study. Koster et al. found that moderate doses of TXA significantly increased the in-hospital mortality during cardiac surgery [37]. However, another study found lower mortality rate in the TXA group [9].

Approximately, 1.2–3.0% of patients undergoing cardiac surgery required postoperative CRRT [38]. Almost 95% of TXA is excreted unchanged through the urine, which typically lasts more than 24 h [2]. There were reports about acute renal failure with thrombosis caused by TXA administration for hemoptysis [39]. We observed that 2.3% of patients needed postoperative CRRT in the TXA group, which was similar to the result of Jakobsen’ study [40], but there was no significant difference between the TXA and non-TXA groups. This finding requires further investigation to confirm in the future, especially for those at high risk in cardiac surgery.

### Limitations

There are several limitations of this investigation. First, it is an observational cohort study; the basic clinical features between the non-TXA group and TXA group were different, such as atrial fibrillation, anticoagulants, redo surgery and transfusion. Although this study applied the propensity score and multivariate regression analysis for reduce evident biases, it is possible that potential confounding variables not included in the analysis may bias the findings. Second, although embolization is another major machanism for postoperative stroke, cardiac surgical patients undergoing CPB share common risks for postoperative outcomes including neurovascular, cardiac, and renal complications. Finally, the low incidence of stroke and relative small sample size of the present study indicate that further large and multicenter studies are needed in this research area of cardiac surgery and anesthesia.

## Conclusions

Based on the 5-year experience of TXA administration in cardiac surgery with CPB, we found that postoperative stroke was associated with intraoperative TXA adminstration in patients undergoing cardiac surgery, especially in those undergoing valve surgeries only. This study may suggest that TXA should be administrated according to clear indications after evaluating the bleeding risk in patients undergoing cardiac surgery, especially in those with high stroke risk.

## Supporting information

S1 Table Demographic and operative characteristics between patients with postoperative complications and not. (DOC)

S2 Table The results of postoperative complications after excluding the patients undergoing CABG surgery. (DOC)

S3 Table After excluding the patients undergoing CABG surgery, demographic and operative characteristics between patients with postoperative complications and not. (DOC)

S1 Fig Number of stroke case during postoperative period. (TIF)

S2 Fig Incidence of postoperative stroke in different surgeries. (TIF)

S1 Text Study data. (XLSX)

S2 Text Study protocol. (DOC)

S3 Text Study IRB in English and in original language. (PDF)
