# Supplementary material for: Intraoperative tranexamic acid is associated with postoperative stroke in patients undergoing cardiac surgery
Source: PLoS One. 2017 May 26;12(5):e0177011. doi: 10.1371/journal.pone.0177011 (PMC5446127; doi:10.1371/journal.pone.0177011)
Supplement: S1 Table — (DOC) [file pone.0177011.s001.doc]

| **S1 Table A.** **Demographic and Clinical characteristics between patients with stroke or not.** | | | | | |
| --- | --- | --- | --- | --- | --- |
| Preoperative Characteristics |  | Stroke | | p-value | |
|  |  | Yes (N= 18) | No (N= 1990) |  |  |
| Age [mean(SD); yr] |  | 60 ± 10 | 52 ± 13 | 0.010 | |
| Male/female, no. (%) |  | 12/6(66.7%) | 941/1049(47.3%) | 0.101 | |
| BMI [mean(SD); kg/m^2^] |  | 22.1 ± 4.5 | 22.3 ± 3.1 | 0.862 | |
| ASA, no. (%) |  |  |  | 0.668 | |
| I |  | 0(0%) | 6(0.3%) |  | |
| II |  | 4(22.2%) | 392(19.7%) |  | |
| III |  | 11(61.1%) | 1437(72.2%) |  | |
| IV |  | 3(16.7%) | 151(7.6%) |  | |
| V |  | 0(0%) | 4(0.2%) |  | |
| NYHA class III/IV, no.(%) |  | 9(50.0%) | 569(28.6%) | 0.046 | |
| History of smoking, no. (%) |  | 9(50.0%) | 474(23.8%) | 0.010 | |
| **Coexistent disease** | | | | | |
| AF, no. (%) |  | 5(27.8%) | 480(24.1%) | 0.718 | |
| Hypertension, no. (%) |  | 7(38.9%) | 460(23.1%) | 0.115 | |
| Diabetes, no. (%) |  | 1(5.6%) | 148(7.4%) | 1# | |
| HLP, no. (%) |  | 0(0%) | 16(0.8%) | 1# | |
| Cerebrovascular disease, no. (%) |  | 4(22.2%) | 113(5.7%) | 0.018# | |
| CKD, no. (%) |  | 1(5.6%) | 34(1.7%) | 0.272# | |
| Liver dysfunction, no. (%) |  | 0(0%) | 20(1.0%) | 1# | |
| COPD, no. (%) |  | 0(0%) | 33(1.7%) | 1# | |
| Infective Endocarditis, no. (%) |  | 0(0%) | 40(2.0%) | 1# | |
| MI, no. (%) |  | 1(5.6%) | 60(3.0%) | 0.427# | |
| Preoperative shock, no. (%) |  | 0(0%) | 2(0.1%) | 1# | |
| Preoperative anemia, no. (%) |  | 2(11.1%) | 272(13.9%) | 1# | |
| #: Fisher’s exact test was used; BMI= body mass index; ASA= American Society of Anesthesiologists; NYHA= New York Heart Association; AF= atrial fibrillation; HLP= Hyperlipidaemia; CKD= chronic kidney disease; COPD= chronic obstructive pulmonary disease; MI= myocardial infarction in 30 days before operation. | | | | |  |

| **Continue S1 Table A. Demographic and Clinical characteristics between patients with stroke or not.** | | | | |
| --- | --- | --- | --- | --- |
| Preoperative Characteristics |  | Stroke | | p-value |
|  |  | Yes (N= 18) | No (N= 1990) |  |
| **Preoperative Medication** | | | | |
| ARB or ACEI, no. (%) |  | 2(11.1%) | 157(7.9%) | 0.648# |
| β-blockers, no.(%) |  | 1(5.6%) | 162(8.1%) | 1# |
| Calcium Channel Blockers, no. (%) |  | 1(5.6%) | 127(6.4%) | 1# |
| Nitrates, no. (%) |  | 2(11.1%) | 85(4.3%) | 0.182# |
| Coumadin, no. (%) |  | 1(5.6%) | 84(4.2%) | 0.542# |
| Heparin, no. (%) |  | 0(0%) | 4(0.2%) | 1# |
| Clopidogrel, no. (%) |  | 3(16.7%) | 76(3.8%) | 0.031# |
| Aspirin, no. (%) |  | 4(22.2%) | 191(9.6%) | 0.089# |
| Statin use, no.(%) |  | 2(11.1%) | 98(4.9%) | 0.225# |
| Diuretics, no. (%) |  | 3(16.7%) | 164(8.2%) | 0.184# |
| Digoxin, no. (%) |  | 1(5.6%) | 103(5.2%) | 0.618# |
| **Preoperative laboratory examination** | | | | |
| LVEF <35%, no.(%) |  | 1(5.6%) | 13(0.7%) | 0.119# |
| T-ch [mean(SD); mmol/L] |  | 4.1 ± 0.7 | 4.3 ± 1.1 | 0.347 |
| BUN [mean(SD); mmol/L] |  | 6.8 ± 1.8 | 6.0 ± 2.3 | 0.145 |
| Hb [mean(SD); g ·L^-1^] |  | 134 ± 14 | 132 ± 19 | 0.643 |
| PLt [mean(SD); 10^3^/mm^3^] |  | 146 ± 48 | 171 ± 58 | 0.073 |
| INR [mean(SD); seconds] |  | 1.08 ± 0.12 | 1.08 ± 0.27 | 0.916 |
| Propensity score [median(SD)] |  | 0.40 ±0.28 | 0.33 ±0.23 | 0.176 |
| #: Fisher’s exact test was used; ARB= angiotensin receptor blockers; ACEI= angiotensin converting enzyme inhibitors; LVEF= left ventricular ejection fraction; T-ch= serum cholesterol; BUN= serum urea nitrogen; Hb= hemoglobin; PLt= Platelet count; INR= international normalized ratio. | | | | |

| **S1 Table B.** **Operative Characteristics between patients with stroke or not** | | | | |
| --- | --- | --- | --- | --- |
| Characteristics |  | Stroke | | p-value |
|  |  | Yes (N= 18) | No (N= 1990) |  |
| Redo surgery |  | 1(5.6%) | 27(1.4%) | 0.224# |
| Emergent operation |  | 1(5.6%) | 18(0.9%) | 0.158# |
| **Type of surgery, no. (%)** |  |  |  | 0.708 |
| CABG Only |  | 3(16.7%) | 211(10.6%) |  |
| Aortic valve |  | 0(0%) | 241(12.1%) |  |
| Mitral valve |  | 3(16.7%) | 365(18.3%) |  |
| Tricuspid valve |  | 1(5.6%) | 97(4.9%) |  |
| Complex cardiac |  | 9(50.0%) | 728(36.6%) |  |
| Transplant |  | 0(0%) | 6(0.3%) |  |
| Aortic |  | 1(5.6%) | 84(4.2%) |  |
| Others |  | 1(5.6%) | 258(13.0%) |  |
| Duration of anesthesia [mean(SD); min] |  | 412 ± 204 | 309 ± 104 | 0.045 |
| Duration of surgery [mean(SD); min] |  | 363 ± 204 | 263 ± 100 | 0.054 |
| CPB time [mean(SD); min] |  | 170 ± 136 | 128 ± 60 | 0.206 |
| IABA, no. (%) |  | 0(0%) | 10(0.5%) | 1# |
| | #: Fisher’s exact test was used; Redo surgery= ≥1 previous sternotomy; CABG= coronary artery bypass grafting; Aortic valve surgery= without ascending aortic replacement; Complex cardiac surgery= combined coronary artery bypass graft surgery and valve surgery or multi-valve surgery; Aortic surgery= Aortic dissections, type A and B, thoracic aortic aneurysms) or Aortic valve surgery with ascending aortic replacement; Others surgery type including ASD (atrial septal defect), VSD (interventricular septal defect), LAM (atrial myxoma), ASV (Aneurysm Sinus Valsalva), CPF (coronary artery pulmonary artery fistula), patent foramen ovale (PFO)/atrial septal aneurysm surgery, and surgery for cardiac tumors; IABA= intra-aortic balloon pump. | | --- | | | | | |

| **Continue S1 Table B. Operative Characteristics between patients with stroke or not** | | | | |
| --- | --- | --- | --- | --- |
| Characteristics |  | Stroke | | p-value |
|  |  | Yes (N= 18) | No (N= 1990) |  |
| **Intra-operative transfusion** |  |  |  |  |
| RBCs transfusion, no. (%) |  | 5(27.8%) | 458(23.0%) | 0.633 |
| FFP transfusion, no. (%) |  | 9(50.0%) | 727(36.5%) | 0.238 |
| Platelet transfusion, no. (%) |  | 3(16.7%) | 177(8.9%) | 0.215# |
| **Input and Output Characteristics** |  |  |  |  |
| Blood loss [mean(SD); mL] |  | 1094 ± 1082 | 672 ± 385 | 0.116 |
| Urine output [mean(SD); mL] |  | 744 ± 378 | 766 ± 491 | 0.854 |
| Pump blood [mean(SD); mL] |  | 467 ± 69 | 516 ± 133 | 0.118 |
| Autologous transfusion [median(IQR); mL] |  | 472(250~563) | 300(200~500) | 0.121 |
| ANH [median(IQR); mL] |  | 0(0~50) | 0(0~0) | 0.815 |
| Introperative Crystals [mean(SD); mL] |  | 1611 ± 557 | 1511 ± 574 | 0.460 |
| Introperative Colloid [mean(SD); mL] |  | 889 ± 471 | 684 ± 373 | 0.021 |
| **Intra-operative coagulation drugs** |  |  |  |  |
| PCC, no. (%) |  | 0(0%) | 58(2.9%) | 1# |
| Fb, no. (%) |  | 0(0%) | 66(3.3%) | 1# |
| rFVIIa, no. (%) |  | 0(0%) | 8(0.4%) | 1# |
| TXA, no. (%) |  | 11(61.1%) | 652(32.8%) | 0.011 |
| | #: Fisher’s exact test was used; RBCs= red blood cells; PCC= Prothrombin Complex Concentrate; Fb= Fibrinogen concentrate; rFVIIa= recombinant activated factor VII; Hb= hemoglobin; Hct= hematocrit; TXA= Tranexamic acid. | | --- | | | | | |

| **S1 Table C. Demographic and Clinical characteristics between patients with seizure or not.** | | | | |
| --- | --- | --- | --- | --- |
| Preoperative Characteristics |  | Seizure | | p-value |
|  |  | Yes (N= 24) | No (N= 1984) |  |
| Age [mean(SD); yr] |  | 54 ± 17 | 52 ± 13 | 0.612 |
| Male/female, no. (%) |  | 14/10(58.3%) | 939/1045(47.3%) | 0.497 |
| BMI [mean(SD); kg/m^2^] |  | 21.9 ± 3.5 | 22.3 ± 3.1 | 0.521 |
| ASA, no. (%) |  |  |  | 0.813 |
| I |  | 0(0%) | 6(0.3) |  |
| II |  | 3(12.5%) | 393(19.8%) |  |
| III |  | 20(83.3%) | 1428(72.0%) |  |
| IV |  | 1(4.2%) | 153(7.7%) |  |
| V |  | 0(0%) | 4(0.2%) |  |
| NYHA class III/IV, no.(%) |  | 8(33.3%) | 570(28.7%) | 0.621 |
| History of smoking, no. (%) |  | 10(41.7%) | 473(23.8%) | 0.042 |
| **Coexistent disease** | | | | |
| AF, no. (%) |  | 4(16.7%) | 481(24.2%) | 0.479# |
| Hypertension, no. (%) |  | 12(50.0%) | 455(22.9%) | 0.002 |
| Diabetes, no. (%) |  | 4(16.7%) | 145(7.3%) | 0.097# |
| HLP, no. (%) |  | 0(0%) | 16(0.8%) | 1# |
| Cerebrovascular disease, no. (%) |  | 3(12.5%) | 114(5.7%) | 0.161# |
| CKD, no. (%) |  | 1(4.2%) | 34(1.7%) | 0.346# |
| Liver dysfunction, no. (%) |  | 0(0%) | 20(1.0%) | 1# |
| COPD, no. (%) |  | 1(4.2%) | 32(1.6%) | 0.330# |
| Infective Endocarditis, no. (%) |  | 0(0%) | 40(2.0%) | 1# |
| MI, no. (%) |  | 2(8.3%) | 59(3.0%) | 0.164# |
| Preoperative shock, no. (%) |  | 0(0%) | 2(0.1%) | 1# |
| Preoperative anemia, no. (%) |  | 3(13.0%) | 271(13.9%) | 1# |
| #: Fisher’s exact test was used; BMI= body mass index; ASA= American Society of Anesthesiologists; NYHA= New York Heart Association; AF= atrial fibrillation; HLP= Hyperlipidaemia; CKD= chronic kidney disease; COPD= chronic obstructive pulmonary disease; MI= myocardial infarction in 30 days before operation. | | | | |

| **Continue S1 Table C. Demographic and Clinical characteristics between patients with seizure or not.** | | | | |
| --- | --- | --- | --- | --- |
| Preoperative Characteristics |  | Seizure | | p-value |
|  |  | Yes (N= 24) | No (N= 1984) |  |
| **Preoperative Medication** | | | | |
| ARB or ACEI, no. (%) |  | 5(20.8%) | 154(7.8%) | 0.018 |
| β-blockers, no.(%) |  | 3(12.5%) | 160(8.1%) | 0.438# |
| Calcium Channel Blockers, no. (%) |  | 2(8.3%) | 126(6.4%) | 0.663# |
| Nitrates, no. (%) |  | 2(8.3%) | 85(4.3%) | 0.279# |
| Coumadin, no. (%) |  | 0(0%) | 85(4.3%) | 0.622# |
| Heparin, no. (%) |  | 0(0%) | 4(0.2%) | 1# |
| Clopidogrel, no. (%) |  | 2(8.3%) | 77(3.9%) | 0.243# |
| Aspirin, no. (%) |  | 3(12.5%) | 192(9.7%) | 0.502# |
| Statin use, no.(%) |  | 1(4.2%) | 99(5.0%) | 1# |
| Diuretics, no. (%) |  | 3(12.5%) | 164(8.3%) | 0.445# |
| Digoxin, no. (%) |  | 0(0%) | 104(5.2%) | 0.633# |
| **Preoperative laboratory examination** | | | | |
| LVEF <35%, no.(%) |  | 1(4.2%) | 13(0.7%) | 0.155# |
| T-ch [mean(SD); mmol/L] |  | 4.4 ± 0.9 | 4.3 ± 1.1 | 0.391 |
| BUN [mean(SD); mmol/L] |  | 6.4 ± 2.4 | 6.0 ± 2.3 | 0.632 |
| Hb [mean(SD); g ·L^-1^] |  | 134 ± 21 | 132 ± 18 | 0.452 |
| PLt [mean(SD); 10^3^/mm^3^] |  | 163 ± 46 | 171 ± 58 | 0.520 |
| INR [mean(SD); seconds] |  | 1.06 ± 0.10 | 1.08 ± 0.27 | 0.660 |
| Propensity score [median(SD)] |  | 0.34 ±0.25 | 0.33 ±0.23 | 0.801 |
| #: Fisher’s exact test was used; ARB= angiotensin receptor blockers; ACEI= angiotensin converting enzyme inhibitors; LVEF= left ventricular ejection fraction; T-ch= serum cholesterol; BUN= serum urea nitrogen; Hb= hemoglobin; PLt= Platelet count; INR= international normalized ratio. | | | | |

| **Supplemental S1 Table D. Operative Characteristics between patients with seizure or not.** | | | | |
| --- | --- | --- | --- | --- |
| Characteristics |  | Seizure | | p-value |
|  |  | Yes (N= 24) | No (N= 1984) |  |
| Redo surgery |  | 0(0%) | 28(1.4%) | 1# |
| Emergent operation |  | 1(4.2%) | 18(0.9%) | 0.205# |
| **Type of surgery, no. (%)** |  |  |  | 0.214 |
| CABG Only |  | 4(16.7%) | 210(10.6%) |  |
| Aortic valve |  | 2(8.3%) | 239(12.0%) |  |
| Mitral valve |  | 9(37.5%) | 359(18.1%) |  |
| Tricuspid valve |  | 1(4.2%) | 97(4.9%) |  |
| Complex cardiac |  | 7(29.2%) | 730(36.8%) |  |
| Transplant |  | 0(0%) | 6(0.3%) |  |
| Aortic |  | 1(4.2%) | 84(4.2%) |  |
| Others |  | 0(0%) | 259(13.1%) |  |
| Duration of anesthesia [mean(SD); min] |  | 361 ± 143 | 309 ± 105 | 0.016 |
| Duration of surgery [mean(SD); min] |  | 329 ± 132 | 263 ± 101 | 0.002 |
| CPB time [mean(SD); min] |  | 165 ± 104 | 128 ± 60 | 0.089 |
| IABA, no. (%) |  | 0(0%) | 10(0.5%) | 1# |
| #: Fisher’s exact test was used; Redo surgery= ≥1 previous sternotomy; CABG= coronary artery bypass grafting; Aortic valve surgery= without ascending aortic replacement; Complex cardiac surgery= combined coronary artery bypass graft surgery and valve surgery or multi-valve surgery; Aortic surgery= Aortic dissections, type A and B, thoracic aortic aneurysms) or Aortic valve surgery with ascending aortic replacement; Others surgery type including ASD (atrial septal defect), VSD (interventricular septal defect), LAM (atrial myxoma), ASV (Aneurysm Sinus Valsalva), CPF (coronary artery pulmonary artery fistula), patent foramen ovale (PFO)/atrial septal aneurysm surgery, and surgery for cardiac tumors; IABA= intra-aortic balloon pump. | | | | |

| **Continue S1 Table D. Operative Characteristics between patients with seizure or not.** | | | | |
| --- | --- | --- | --- | --- |
| Characteristics |  | Seizure | | p-value |
|  |  | Yes (N= 24) | No (N= 1984) |  |
| **Intra-operative transfusion** |  |  |  |  |
| RBCs transfusion, no. (%) |  | 7(29.2%) | 456(23.0%) | 0.475 |
| FFP transfusion, no. (%) |  | 10(41.7%) | 726(36.6%) | 0.608 |
| Platelet transfusion, no. (%) |  | 4(16.7%) | 176(8.9%) | 0.266# |
| **Input and Output Characteristics** |  |  |  |  |
| Blood loss [mean(SD); mL] |  | 675 ± 361 | 676 ± 340 | 0.991 |
| Urine output [mean(SD); mL] |  | 784 ± 498 | 765 ± 490 | 0.854 |
| Pump blood [mean(SD); mL] |  | 558 ± 143 | 515 ± 133 | 0.112 |
| Autologous transfusion [median(IQR); mL] |  | 333(163~488) | 300(200~500) | 0.808 |
| ANH [median(IQR); mL] |  | 0(0~0) | 0(0~0) | 0.093 |
| Introperative Crystals [mean(SD); mL] |  | 1563 ± 727 | 1511 ± 572 | 0.663 |
| Introperative Colloid [mean(SD); mL] |  | 792 ± 487 | 685 ± 373 | 0.296 |
| **Intraoperative coagulation drugs** |  |  |  |  |
| PCC, no. (%) |  | 2(8.3%) | 56(2.8%) | 0.151# |
| Fb, no. (%) |  | 1(4.2%) | 65(3.3%) | 0.554# |
| rFVIIa, no. (%) |  | 0(0%) | 8(0.4%) | 1# |
| TXA, no. (%) |  | 8(33.3%) | 655(33.0%) | 0.974 |
| | #: Fisher’s exact test was used; RBCs= red blood cells; PCC= Prothrombin Complex Concentrate; Fb= Fibrinogen concentrate; rFVIIa= recombinant activated factor VII; Hb= hemoglobin; Hct= hematocrit; TXA= Tranexamic acid. | | --- | | | | | |

| **S1 Table E. Demographic and Clinical characteristics between patients with coma or not.** | | | | | |
| --- | --- | --- | --- | --- | --- |
| Preoperative Characteristics |  | Coma | | p-value | |
|  |  | Yes (N= 25) | No (N= 1983) |  |  |
| Age [mean(SD); yr] |  | 56 ± 13 | 52 ± 13 | 0.111 | |
| Male/female, no. (%) |  | 17/8(68.0%) | 936/1047(47.2%) | 0.038 | |
| BMI [mean(SD); kg/m^2^] |  | 21.9 ± 3.9 | 22.3 ± 3.1 | 0.486 | |
| ASA, no. (%) |  |  |  | 0.226 | |
| I |  | 0(0%) | 6(0.3%) |  | |
| II |  | 5(20.0%) | 391(19.7%) |  | |
| III |  | 15(60.0%) | 1433(72.3%) |  | |
| IV |  | 5(20.0%) | 149(7.5%) |  | |
| V |  | 0(0%) | 4(0.2%) |  | |
| NYHA class III/IV, no.(%) |  | 12(48.0%) | 566(28.5%) | 0.033 | |
| History of smoking, no. (%) |  | 10(40.0%) | 473(23.9%) | 0.060 | |
| **Coexistent disease** | | | | | |
| AF, no. (%) |  | 5(20.0%) | 480(24.2%) | 0.625 | |
| Hypertension, no. (%) |  | 10(40.0%) | 457(23.0%) | 0.046 | |
| Diabetes, no. (%) |  | 2(8.0%) | 147(7.4%) | 0.708# | |
| HLP, no. (%) |  | 0(0%) | 16(0.8%) | 1# | |
| Cerebrovascular disease, no. (%) |  | 5(20.0%) | 112(5.6%) | 0.002 | |
| CKD, no. (%) |  | 2(8.0%) | 33(1.7%) | 0.069# | |
| Liver dysfunction, no. (%) |  | 1(4.0%) | 19(1.0%) | 0.223# | |
| COPD, no. (%) |  | 0(0%) | 33(1.7%) | 1# | |
| Infective Endocarditis, no. (%) |  | 0(0%) | 40(2.0%) | 1# | |
| MI, no. (%) |  | 1(4.0%) | 60(3.0%) | 0.540# | |
| Preoperative shock, no. (%) |  | 0(0%) | 2(0.1%) | 1# | |
| Preoperative anemia, no. (%) |  | 4(16.0%) | 270(13.9%) | 0.769# | |
| #: Fisher’s exact test was used; BMI= body mass index; ASA= American Society of Anesthesiologists; NYHA= New York Heart Association; AF= atrial fibrillation; HLP= Hyperlipidaemia; CKD= chronic kidney disease; COPD= chronic obstructive pulmonary disease; MI= myocardial infarction in 30 days before operation. | | | | |  |

| **Continue S1 Table E. Demographic and Clinical characteristics between patients with coma or not.** | | | | |
| --- | --- | --- | --- | --- |
| Preoperative Characteristics |  | Coma | | p-value |
|  |  | Yes (N= 25) | No (N= 1983) |  |
| **Preoperative Medication** | | | | |
| ARB or ACEI, no. (%) |  | 3(12.0%) | 156(7.9%) | 0.443# |
| β-blockers, no.(%) |  | 1(4.0%) | 162(8.2%) | 0.716# |
| Calcium Channel Blockers, no. (%) |  | 2(8.0%) | 126(6.4%) | 0.671# |
| Nitrates, no. (%) |  | 2(8.0%) | 85(4.3%) | 0.296# |
| Coumadin, no. (%) |  | 2(8.0%) | 83(4.2%) | 0.286# |
| Heparin, no. (%) |  | 0(0%) | 4(0.2%) | 1# |
| Clopidogrel, no. (%) |  | 3(12.0%) | 76(3.8%) | 0.072# |
| Aspirin, no. (%) |  | 4(16.0%) | 191(9.6%) | 0.296# |
| Statin use, no.(%) |  | 3(12.0%) | 97(4.9%) | 0.125# |
| Diuretics, no. (%) |  | 5(20.0%) | 162(8.2%) | 0.033 |
| Digoxin, no. (%) |  | 1(4.0%) | 103(5.2%) | 1# |
| **Preoperative laboratory examination** | | | | |
| LVEF <35%, no.(%) |  | 1(4.0%) | 14(0.7%) | 0.161# |
| T-ch [mean(SD); mmol/L] |  | 3.9 ± 0.8 | 4.3 ± 1.1 | 0.077 |
| BUN [mean(SD); mmol/L] |  | 7.2 ± 4.3 | 6.0 ± 2.2 | 0.156 |
| Hb [mean(SD); g ·L^-1^] |  | 133 ± 18 | 132 ± 18 | 0.727 |
| PLt [mean(SD); 10^3^/mm^3^] |  | 139 ± 46 | 171 ± 58 | 0.007 |
| INR [mean(SD); seconds] |  | 1.10 ± 0.14 | 1.08 ± 0.27 | 0.764 |
| Propensity score [median(SD)] |  | 0.36 ±0.26 | 0.33 ±0.23 | 0.577 |
| #: Fisher’s exact test was used; ARB= angiotensin receptor blockers; ACEI= angiotensin converting enzyme inhibitors; LVEF= left ventricular ejection fraction; T-ch= serum cholesterol; BUN= serum urea nitrogen; Hb= hemoglobin; PLt= Platelet count; INR= international normalized ratio. | | | | |

| **S1 Table F. Operative Characteristics between patients with coma or not** | | | | |
| --- | --- | --- | --- | --- |
| Characteristics |  | Coma | | p-value |
|  |  | Yes (N= 25) | No (N= 1983) |  |
| Redo surgery |  | 1(4.0%) | 27(1.4%) | 0.298# |
| Emergent operation |  | 2(8.0%) | 17(0.9%) | 0.022# |
| **Type of surgery, no. (%)** |  |  |  | 0.131 |
| CABG Only |  | 3(12.0%) | 211(10.6%) |  |
| Aortic valve |  | 1(4.0%) | 240(12.1%) |  |
| Mitral valve |  | 3(12.0%) | 365(18.4%) |  |
| Tricuspid valve |  | 1(4.0%) | 97(4.9%) |  |
| Complex cardiac |  | 11(44.0%) | 726(36.6%) |  |
| Transplant |  | 0(0%) | 6(0.3%) |  |
| Aortic |  | 4(16.0%) | 81(4.1%) |  |
| Others |  | 2(8.0%) | 257(13.0%) |  |
| Duration of anesthesia [mean(SD); min] |  | 429 ± 189 | 308 ± 103 | 0.004 |
| Duration of surgery [mean(SD); min] |  | 380 ± 188 | 263 ± 99 | 0.005 |
| CPB time [mean(SD); min] |  | 187 ± 127 | 128 ± 59 | 0.027 |
| IABA, no. (%) |  | 0(0%) | 10(0.5%) | 1# |
| #: Fisher’s exact test was used; Redo surgery= ≥1 previous sternotomy; CABG= coronary artery bypass grafting; Aortic valve surgery= without ascending aortic replacement; Complex cardiac surgery= combined coronary artery bypass graft surgery and valve surgery or multi-valve surgery; Aortic surgery= Aortic dissections, type A and B, thoracic aortic aneurysms) or Aortic valve surgery with ascending aortic replacement; Others surgery type including ASD (atrial septal defect), VSD (interventricular septal defect), LAM (atrial myxoma), ASV (Aneurysm Sinus Valsalva), CPF (coronary artery pulmonary artery fistula), patent foramen ovale (PFO)/atrial septal aneurysm surgery, and surgery for cardiac tumors; IABA= intra-aortic balloon pump. | | | | |

| **Continue S1 Table F. Operative Characteristics between patients with coma or not** | | | | |
| --- | --- | --- | --- | --- |
| Characteristics |  | Coma | | p-value |
|  |  | Yes (N= 25) | No (N= 1983) |  |
| **Intra-operative transfusion** |  |  |  |  |
| RBCs transfusion, no. (%) |  | 10(40.0%) | 453(22.8%) | 0.043 |
| FFP transfusion, no. (%) |  | 15(60.0%) | 721(36.4%) | 0.015 |
| Platelet transfusion, no. (%) |  | 4(16.0%) | 176(8.9%) | 0.274# |
| **Input and Output Characteristics** |  |  |  |  |
| Blood loss [mean(SD); mL] |  | 1072 ± 1031 | 671 ± 382 | 0.064 |
| Urine output [mean(SD); mL] |  | 768 ± 468 | 766 ± 490 | 0.977 |
| Pump blood [mean(SD); mL] |  | 476 ± 78 | 516 ± 134 | 0.136 |
| Autologous transfusion [median(IQR); mL] |  | 474(250~750) | 300(200~500) | 0.032 |
| ANH [median(IQR); mL] |  | 0(0~0) | 0(0~0) | 0.643 |
| Introperative Crystals [mean(SD); mL] |  | 1680 ± 518 | 1510 ± 574 | 0.140 |
| Introperative Colloid [mean(SD); mL] |  | 788 ± 446 | 685 ± 374 | 0.172 |
| **Intra-operative coagulation drugs** |  |  |  |  |
| PCC, no. (%) |  | 1(4.0%) | 57(2.9%) | 0.522# |
| Fb, no. (%) |  | 1(4.0%) | 65(3.3%) | 0.569# |
| rFVIIa, no. (%) |  | 1(4.0%) | 7(0.4%) | 0.096# |
| TXA, no. (%) |  | 12(48.0%) | 651(32.8%) | 0.109 |
| | #: Fisher’s exact test was used; RBCs= red blood cells; PCC= Prothrombin Complex Concentrate; Fb= Fibrinogen concentrate; rFVIIa= recombinant activated factor VII; Hb= hemoglobin; Hct= hematocrit; TXA= Tranexamic acid. | | --- | | | | | |

| **S1 Table G. Demographic and Clinical characteristics between patients with death or not.** | | | | | |
| --- | --- | --- | --- | --- | --- |
| Preoperative Characteristics |  | Death | | p-value | |
|  |  | Yes (N= 37) | No (N= 1971) |  |  |
| Age [mean(SD); yr] |  | 63 ± 14 | 52 ± 13 | <0.001 | |
| Male/female, no. (%) |  | 14/23(37.8%) | 939/1032(47.6%) | 0.237 | |
| BMI [mean(SD); kg/m^2^] |  | 21.5 ± 3.2 | 22.3 ± 3.1 | 0.102 | |
| ASA, no. (%) |  |  |  | <0.001 | |
| I |  | 0(0%) | 6(0.3%) |  | |
| II |  | 0(0%) | 396(20.1%) |  | |
| III |  | 29(78.4%) | 1419(72.0%) |  | |
| IV |  | 6(16.2%) | 148(7.5%) |  | |
| V |  | 2(5.4%) | 2(0.1%) |  | |
| NYHA class III/IV, no.(%) |  | 18(48.6%) | 560(28.4%) | 0.007 | |
| History of smoking, no. (%) |  | 8(21.6%) | 475(24.1%) | 0.727 | |
| **Coexistent disease** | | | | | |
| AF, no. (%) |  | 12(32.4%) | 473(24.0%) | 0.235 | |
| Hypertension, no. (%) |  | 19(51.4%) | 448(22.7%) | <0.001 | |
| Diabetes, no. (%) |  | 8(21.6%) | 141(7.2%) | 0.001 | |
| HLP, no. (%) |  | 0(0%) | 16(0.8%) | 1# | |
| Cerebrovascular disease, no. (%) |  | 5(13.5%) | 112(5.7%) | 0.044 | |
| CKD, no. (%) |  | 4(10.8%) | 31(1.6%) | 0.003# | |
| Liver dysfunction, no. (%) |  | 0(0%) | 20(1.0%) | 1# | |
| COPD, no. (%) |  | 0(0%) | 33(1.7%) | 1# | |
| Infective Endocarditis, no. (%) |  | 0(0%) | 40(2.0%) | 1# | |
| MI, no. (%) |  | 5(13.5%) | 56(2.8%) | <0.001 | |
| Preoperative shock, no. (%) |  | 0(0%) | 2(0.1%) | 1# | |
| Preoperative anemia, no. (%) |  | 13(36.1%) | 261(13.5%) | <0.001 | |
| #: Fisher’s exact test was used; BMI= body mass index; ASA= American Society of Anesthesiologists; NYHA= New York Heart Association; AF= atrial fibrillation; HLP= Hyperlipidaemia; CKD= chronic kidney disease; COPD= chronic obstructive pulmonary disease; MI= myocardial infarction in 30 days before operation. | | | | |  |

| **Continue S1 Table G. Demographic and Clinical characteristics between patients with death or not.** | | | | |
| --- | --- | --- | --- | --- |
| Preoperative Characteristics |  | Death | | p-value |
|  |  | Yes (N= 37) | No (N= 1971) |  |
| **Preoperative Medication** | | | | |
| ARB or ACEI, no. (%) |  | 6(16.2%) | 153(7.8%) | 0.059 |
| β-blockers, no.(%) |  | 7(18.9%) | 156(7.9%) | 0.015 |
| Calcium Channel Blockers, no. (%) |  | 3(8.1%) | 125(6.3%) | 0.509# |
| Nitrates, no. (%) |  | 6(16.2%) | 81(4.1%) | < 0.001 |
| Coumadin, no. (%) |  | 0(0%) | 85(4.3%) | 0.403# |
| Heparin, no. (%) |  | 0(0%) | 4(0.2%) | 1# |
| Clopidogrel, no. (%) |  | 4(10.8%) | 75(3.8%) | 0.055# |
| Aspirin, no. (%) |  | 8(21.6%) | 187(9.5%) | 0.014 |
| Statin use, no.(%) |  | 6(16.2%) | 94(4.8%) | 0.002 |
| Diuretics, no. (%) |  | 7(18.9%) | 160(8.1%) | 0.018 |
| Digoxin, no. (%) |  | 3(8.1%) | 101(5.1%) | 0.437# |
| **Preoperative laboratory examination** | | | | |
| LVEF <35%, no.(%) |  | 0(0%) | 14(0.7%) | 1# |
| T-ch [mean(SD); mmol/L] |  | 4.0 ± 1.0 | 4.3 ± 1.1 | 0.087 |
| BUN [mean(SD); mmol/L] |  | 8.0 ± 5.2 | 5.9 ± 2.1 | 0.020 |
| Hb [mean(SD); g ·L^-1^] |  | 124 ± 26 | 132 ± 18 | 0.064 |
| PLt [mean(SD); 10^3^/mm^3^] |  | 157 ± 57 | 171 ± 58 | 0.140 |
| INR [mean(SD); seconds] |  | 1.06 ± 0.14 | 1.08 ± 0.27 | 0.527 |
| Propensity score [median(SD)] |  | 0.39 ±0.27 | 0.33 ±0.23 | 0.181 |
| #: Fisher’s exact test was used; ARB= angiotensin receptor blockers; ACEI= angiotensin converting enzyme inhibitors; LVEF= left ventricular ejection fraction; T-ch= serum cholesterol; BUN= serum urea nitrogen; Hb= hemoglobin; PLt= Platelet count; INR= international normalized ratio. | | | | |

| **S1 Table H. Operative Characteristics between patients with death or not.** | | | | |
| --- | --- | --- | --- | --- |
| Characteristics |  | Death | | p-value |
|  |  | Yes (N= 37) | No (N= 1971) |  |
| Redo surgery |  | 2(5.4%) | 26(1.3%) | 0.093# |
| Emergent operation |  | 1(2.7%) | 18(0.9%) | 0.299# |
| **Type of surgery, no. (%)** |  |  |  | 0.018 |
| CABG Only |  | 9(24.3%) | 205(10.4%) |  |
| Aortic valve |  | 2(5.4%) | 239(12.1%) |  |
| Mitral valve |  | 4(10.8%) | 364(18.5%) |  |
| Tricuspid valve |  | 1(2.7%) | 97(4.9%) |  |
| Complex cardiac |  | 16(43.2%) | 721(36.6%) |  |
| Transplant |  | 0(0%) | 6(0.3%) |  |
| Aortic |  | 4(10.%) | 81(4.1%) |  |
| Others |  | 1(2.7%) | 258(13.1%) |  |
| Duration of anesthesia [mean(SD); min] |  | 481 ± 212 | 306 ± 100 | <0.001 |
| Duration of surgery [mean(SD); min] |  | 423 ± 199 | 261 ± 97 | <0.001 |
| CPB time [mean(SD); min] |  | 180 ± 105 | 127 ± 59 | 0.005 |
| IABA, no. (%) |  | 5(13.5%) | 5(0.3%) | <0.001 |
| | #: Fisher’s exact test was used; Redo surgery= ≥1 previous sternotomy; CABG= coronary artery bypass grafting; Aortic valve surgery= without ascending aortic replacement; Complex cardiac surgery= combined coronary artery bypass graft surgery and valve surgery or multi-valve surgery; Aortic surgery= Aortic dissections, type A and B, thoracic aortic aneurysms) or Aortic valve surgery with ascending aortic replacement; Others surgery type including ASD (atrial septal defect), VSD (interventricular septal defect), LAM (atrial myxoma), ASV (Aneurysm Sinus Valsalva), CPF (coronary artery pulmonary artery fistula), patent foramen ovale (PFO)/atrial septal aneurysm surgery, and surgery for cardiac tumors; IABA= intra-aortic balloon pump. | | --- | | | | | |

| **Continue S1 Table H. Operative Characteristics between patients with death or not.** | | | | |
| --- | --- | --- | --- | --- |
| Characteristics |  | Death | | p-value |
|  |  | Yes (N= 37) | No (N= 1971) |  |
| **Intra-operative transfusion** |  |  |  |  |
| RBCs transfusion, no. (%) |  | 23(62.2%) | 440(22.3%) | <0.001 |
| FFP transfusion, no. (%) |  | 29(78.4%) | 707(35.9%) | <0.001 |
| Platelet transfusion, no. (%) |  | 13(35.1%) | 167(8.5%) | <0.001 |
| **Input and Output Characteristics** |  |  |  |  |
| Blood loss [mean(SD); mL] |  | 1135 ± 849 | 667 ± 380 | 0.002 |
| Urine output [mean(SD); mL] |  | 946 ± 542 | 762 ± 488 | 0.024 |
| Pump blood [mean(SD); mL] |  | 534 ± 120 | 515 ± 133 | 0.381 |
| Autologous transfusion [median(IQR); mL] |  | 500(225~750) | 300(200~500) | 0.003 |
| ANH [median(IQR); mL] |  | 0(0~0) | 0(0~0) | 0.289 |
| Introperative Crystals [mean(SD); mL] |  | 1776 ± 854 | 1507 ± 567 | 0.064 |
| Introperative Colloid [mean(SD); mL] |  | 973 ± 565 | 681 ± 368 | 0.003 |
| **Intraoperative coagulation drugs** |  |  |  |  |
| PCC, no. (%) |  | 1(2.7%) | 57(2.9%) | 1# |
| Fb, no. (%) |  | 1(2.7%) | 65(3.3%) | 1# |
| rFVIIa, no. (%) |  | 2(5.4%) | 6(0.3%) | 0.009# |
| TXA, no. (%) |  | 11(29.7%) | 652(33.1%) | 0.668 |
| | #: Fisher’s exact test was used; RBCs= red blood cells; PCC= Prothrombin Complex Concentrate; Fb= Fibrinogen concentrate; rFVIIa= recombinant activated factor VII; Hb= hemoglobin; Hct= hematocrit; TXA= Tranexamic acid. | | --- | | | | | |

| **S1 Table I. Demographic and Clinical characteristics between patients with CRRT or not.** | | | | |
| --- | --- | --- | --- | --- |
| Preoperative Characteristics |  | CRRT | | p-value |
|  |  | Yes (N= 36) | No (N= 1972) |  |
| Age [mean(SD); yr] |  | 62 ± 14 | 52 ± 13 | <0.001 |
| Male/female, no. (%) |  | 19/17(52.8%) | 934/1038(47.4%) | 0.519 |
| BMI [mean(SD); kg/m^2^] |  | 22.1 ± 3.6 | 22.3 ± 3.1 | 0.704 |
| ASA, no. (%) |  |  |  | 0.002 |
| I |  | 0(0%) | 6(0.3%) |  |
| II |  | 3(8.3%) | 393(19.9%) |  |
| III |  | 27(75.0%) | 1421(72.1%) |  |
| IV |  | 5(13.9%) | 149(7.6%) |  |
| V |  | 1(2.8%) | 3(0.2%) |  |
| NYHA class III/IV, no.(%) |  | 15(41.7%) | 563(28.5%) | 0.085 |
| History of smoking, no. (%) |  | 12(33.3%) | 471(23.9%) | 0.189 |
| **Coexistent disease** | | | | |
| AF, no. (%) |  | 10(27.8%) | 475(24.1%) | 0.608 |
| Hypertension, no. (%) |  | 20(55.6%) | 447(22.7%) | <0.001 |
| Diabetes, no. (%) |  | 7(19.4%) | 142(7.2%) | 0.005 |
| HLP, no. (%) |  | 0(0%) | 16(0.8%) | 1# |
| Cerebrovascular disease, no. (%) |  | 8(22.2%) | 109(5.5%) | <0.001 |
| CKD, no. (%) |  | 5(13.9%) | 30(1.5%) | <0.001 |
| Liver dysfunction, no. (%) |  | 0(0%) | 20(1.0%) | 1# |
| COPD, no. (%) |  | 0(0%) | 33(1.7%) | 1# |
| Infective Endocarditis, no. (%) |  | 0(0%) | 40(2.0%) | 1# |
| MI, no. (%) |  | 2(5.6%) | 59(3.0%) | 0.299# |
| Preoperative shock, no. (%) |  | 0(0%) | 2(0.1%) | 1# |
| Preoperative anemia, no. (%) |  | 13(36.1%) | 261(13.5%) | < 0.001 |
| #: Fisher’s exact test was used; BMI= body mass index; ASA= American Society of Anesthesiologists; NYHA= New York Heart Association; AF= atrial fibrillation; HLP= Hyperlipidaemia; CKD= chronic kidney disease; COPD= chronic obstructive pulmonary disease; MI= myocardial infarction in 30 days before operation; CRRT= continuous renal replacement therapy. | | | | |

| **Continue S1 Table I. Demographic and Clinical characteristics between patients with CRRT or not.** | | | | |
| --- | --- | --- | --- | --- |
| Preoperative Characteristics |  | CRRT | | p-value |
|  |  | Yes (N= 36) | No (N= 1972) |  |
| **Preoperative Medication** | | | | |
| ARB or ACEI, no. (%) |  | 6(16.7%) | 153(47.8%) | 0.050 |
| β-blockers, no.(%) |  | 6(16.7%) | 157(8.0%) | 0.058 |
| Calcium Channel Blockers, no. (%) |  | 4(11.1%) | 124(6.3%) | 0.285# |
| Nitrates, no. (%) |  | 5(13.9%) | 82(4.2%) | 0.004 |
| Coumadin, no. (%) |  | 0(0%) | 85(4.3%) | 0.401# |
| Heparin, no. (%) |  | 0(0%) | 4(0.2%) | 1# |
| Clopidogrel, no. (%) |  | 4(11.1%) | 75(3.8%) | 0.050# |
| Aspirin, no. (%) |  | 8(22.2%) | 187(9.5%) | 0.011 |
| Statin use, no.(%) |  | 3(8.3%) | 97(4.9%) | 0.422# |
| Diuretics, no. (%) |  | 4(11.1%) | 163(8.3%) | 0.536# |
| Digoxin, no. (%) |  | 4(11.1%) | 100(5.1%) | 0.112# |
| **Preoperative laboratory examination** | | | | |
| LVEF <35%, no.(%) |  | 1(2.8%) | 13(0.7%) | 0.224# |
| T-ch [mean(SD); mmol/L] |  | 4.2 ± 1.0 | 4.3 ± 1.1 | 0.637 |
| BUN [mean(SD); mmol/L] |  | 8.1 ± 5.1 | 5.9 ± 2.2 | 0.017 |
| Hb [mean(SD); g ·L^-1^] |  | 122 ± 23 | 132 ± 18 | 0.019 |
| PLt [mean(SD); 10^3^/mm^3^] |  | 167 ± 58 | 171 ± 58 | 0.703 |
| INR [mean(SD); seconds] |  | 1.18 ± 0.46 | 1.08 ± 0.27 | 0.187 |
| Propensity score [median(SD)] |  | 0.45 ±0.31 | 0.33 ±0.23 | 0.023 |
| #: Fisher’s exact test was used; ARB= angiotensin receptor blockers; ACEI= angiotensin converting enzyme inhibitors; LVEF= left ventricular ejection fraction; T-ch= serum cholesterol; BUN= serum urea nitrogen; Hb= hemoglobin; PLt= Platelet count; INR= international normalized ratio. | | | | |

| **S1 Table J. Operative Characteristics between patients with CRRT or not.** | | | | |
| --- | --- | --- | --- | --- |
| Characteristics |  | CRRT | | p-value |
|  |  | Yes (N= 36) | No (N= 1972) |  |
| Redo surgery |  | 2(5.6%) | 26(1.3%) | 0.088# |
| Emergent operation |  | 2(5.6%) | 17(.9%) | 0.044# |
| **Type of surgery, no. (%)** |  |  |  | 0.370 |
| CABG Only |  | 7(19.4%) | 207(10.5%) |  |
| Aortic valve |  | 4(11.1%) | 237(12.0%) |  |
| Mitral valve |  | 7(19.4%) | 361(18.3%) |  |
| Tricuspid valve |  | 0(0%) | 98(5.0%) |  |
| Complex cardiac |  | 13(36.1%) | 724(36.7%) |  |
| Transplant |  | 0(0%) | 6(0.3%) |  |
| Aortic |  | 3(8.3%) | 82(4.2%) |  |
| Others |  | 2(5.6%) | 257(13.0%) |  |
| Duration of anesthesia [mean(SD); min] |  | 435 ± 217 | 307 ± 101 | 0.001 |
| Duration of surgery [mean(SD); min] |  | 364 ± 203 | 262 ± 98 | 0.005 |
| CPB time [mean(SD); min] |  | 175 ± 140 | 127 ± 58 | 0.050 |
| IABA, no. (%) |  | 4(11.1%) | 6(0.3%) | <0.001 |
| | #: Fisher’s exact test was used; Redo surgery= ≥1 previous sternotomy; CABG= coronary artery bypass grafting; Aortic valve surgery= without ascending aortic replacement; Complex cardiac surgery= combined coronary artery bypass graft surgery and valve surgery or multi-valve surgery; Aortic surgery= Aortic dissections, type A and B, thoracic aortic aneurysms) or Aortic valve surgery with ascending aortic replacement; Others surgery type including ASD (atrial septal defect), VSD (interventricular septal defect), LAM (atrial myxoma), ASV (Aneurysm Sinus Valsalva), CPF (coronary artery pulmonary artery fistula), patent foramen ovale (PFO)/atrial septal aneurysm surgery, and surgery for cardiac tumors; IABA= intra-aortic balloon pump. | | --- | | | | | |

| **Continue S1 Table J. Operative Characteristics between patients with CRRT or not.** | | | | |
| --- | --- | --- | --- | --- |
| Characteristics |  | CRRT | | p-value |
|  |  | Yes (N= 36) | No (N= 1972) |  |
| **Intra-operative transfusion** |  |  |  |  |
| RBCs transfusion, no. (%) |  | 16(44.4%) | 447(22.7%) | 0.002 |
| FFP transfusion, no. (%) |  | 23(63.9%) | 713(36.2%) | 0.001 |
| Platelet transfusion, no. (%) |  | 9(25.0%) | 171(8.7%) | 0.001 |
| **Input and Output Characteristics** |  |  |  |  |
| Blood loss [mean(SD); mL] |  | 1094 ± 908 | 668 ± 379 | 0.008 |
| Urine output [mean(SD); mL] |  | 862 ± 637 | 764 ± 487 | 0.366 |
| Pump blood [mean(SD); mL] |  | 523 ± 117 | 515 ± 133 | 0.735 |
| Autologous transfusion [median(IQR); mL] |  | 400(202~600) | 300(200~500) | 0.248 |
| ANH [median(IQR); mL] |  | 0(0~0) | 0(0~0) | 0.229 |
| Introperative Crystals [mean(SD); mL] |  | 1556 ± 684 | 1511 ± 572 | 0.644 |
| Introperative Colloid [mean(SD); mL] |  | 931 ± 599 | 682 ± 368 | 0.018 |
| **Intra-operative coagulation drugs** |  | 449 ± 224 | 311 ± 104 | 0.003 |
| PCC, no. (%) |  | 2(5.6%) | 56(2.8%) | 0.279# |
| Fb, no. (%) |  | 2(5.6%) | 64(3.2%) | 0.333# |
| rFVIIa, no. (%) |  | 2(5.6%) | 6(0.3%) | 0.008# |
| TXA, no. (%) |  | 14(38.9%) | 649(32.9%) | 0.450 |
| | #: Fisher’s exact test was used; RBCs= red blood cells; PCC= Prothrombin Complex Concentrate; Fb= Fibrinogen concentrate; rFVIIa= recombinant activated factor VII; Hb= hemoglobin; Hct= hematocrit; TXA= Tranexamic acid. | | --- | | | | | |

| **S1 Table K. Demographic and Clinical characteristics between patients with** **resternotomy for postoperative bleeding or not.** | | | | | |
| --- | --- | --- | --- | --- | --- |
| Preoperative Characteristics |  | Resternotomy for  postoperative bleeding | | p-value | |
|  |  | Yes (N= 26) | No (N= 1982) |  |  |
| Age [mean(SD); yr] |  | 57 ± 14 | 52 ± 13 | 0.047 | |
| Male/female, no. (%) |  | 13/13(50.0%) | 940/1042(47.4%) | 0.794 | |
| BMI [mean(SD); kg/m^2^] |  | 21.5 ± 3.1 | 22.3 ± 3.1 | 0.201 | |
| ASA, no. (%) |  |  |  | 0.005 | |
| I |  | 0(0%) | 6(0.3%) |  | |
| II |  | 2(7.7%) | 394(19.9%) |  | |
| III |  | 17(65.4%) | 1431(72.2%) |  | |
| IV |  | 7(26.9%) | 147(7.4%) |  | |
| V |  | 0(0%) | 4(0.2%) |  | |
| NYHA class III/IV, no.(%) |  | 14(53.8%) | 564(28.5%) | 0.004 | |
| History of smoking, no. (%) |  | 8(30.8%) | 475(24.0%) | 0.420 | |
| **Coexistent disease** | | | | | |
| AF, no. (%) |  | 10(38.5%) | 475(24.0%) | 0.086 | |
| Hypertension, no. (%) |  | 7(26.9%) | 460(23.2%) | 0.656 | |
| Diabetes, no. (%) |  | 4(15.4%) | 148(7.3%) | 0.122# | |
| HLP, no. (%) |  | 0(0%) | 16(0.8%) | 1# | |
| Cerebrovascular disease, no. (%) |  | 1(3.8%) | 116(5.9%) | 1# | |
| CKD, no. (%) |  | 0(0%) | 35(1.8%) | 1# | |
| Liver dysfunction, no. (%) |  | 0(0%) | 20(1.0%) | 1# | |
| COPD, no. (%) |  | 2(7.7%) | 31(1.6%) | 0.067# | |
| Infective Endocarditis, no. (%) |  | 0(0%) | 40(2.0%) | 1# | |
| MI, no. (%) |  | 3(11.5%) | 58(2.9%) | 0.042# | |
| Preoperative shock, no. (%) |  | 1(3.8%) | 1(0.1%) | 0.026# | |
| Preoperative anemia, no. (%) |  | 3(11.5%) | 271(13.9%) | 1# | |
| #: Fisher’s exact test was used; BMI= body mass index; ASA= American Society of Anesthesiologists; NYHA= New York Heart Association; AF= atrial fibrillation; HLP= Hyperlipidaemia; CKD= chronic kidney disease; COPD= chronic obstructive pulmonary disease; MI= myocardial infarction in 30 days before operation. | | | | |  |

| **Continue S1 Table K. Demographic and Clinical characteristics between patients with resternotomy for postoperative bleeding or not.** | | | | | |
| --- | --- | --- | --- | --- | --- |
| Preoperative Characteristics |  | | Resternotomy for postoperative bleeding | | p-value |
|  |  | | Yes (N= 26) | No (N= 1982) |  |
| **Preoperative Medication** | | | | | |
| ARB or ACEI, no. (%) | |  | 4(15.4%) | 155(7.8%) | 0.145# |
| β-blockers, no.(%) | |  | 2(7.7%) | 161(8.1%) | 1# |
| Calcium Channel Blockers, no. (%) | |  | 3(11.5%) | 125(6.3%) | 0.228# |
| Nitrates, no. (%) | |  | 4(15.4%) | 83(4.2%) | 0.024# |
| Coumadin, no. (%) | |  | 2(7.7%) | 83(4.2%) | 0.302# |
| Heparin, no. (%) | |  | 0(0%) | 4(0.2%) | 1# |
| Clopidogrel, no. (%) | |  | 1(3.8%) | 78(3.9%) | 1# |
| Aspirin, no. (%) | |  | 2(7.7%) | 193(9.7%) | 1# |
| Statin use, no.(%) | |  | 2(7.7%) | 98(4.9%) | 0.375# |
| Diuretics, no. (%) | |  | 5(19.2%) | 162(8.2%) | 0.043 |
| Digoxin, no. (%) | |  | 4(15.4%) | 100(5.0%) | 0.042# |
| **Preoperative laboratory examination** | | | | | |
| LVEF <35%, no.(%) | |  | 0(0%) | 14(0.7%) | 1# |
| T-ch [mean(SD); mmol/L] | |  | 4.1 ± 0.8 | 4.3 ± 1.1 | 0.311 |
| BUN [mean(SD); mmol/L] | |  | 6.1 ± 2.1 | 6.0 ± 2.3 | 0.713 |
| Hb [mean(SD); g ·L^-1^] | |  | 134 ± 20 | 132 ± 18 | 0.452 |
| PLt [mean(SD); 10^3^/mm^3^] | |  | 156 ± 48 | 171 ± 58 | 0.193 |
| INR [mean(SD); seconds] | |  | 1.14 ± 0.38 | 1.08 ± 0.27 | 0.440 |
| Propensity score [median(SD)] | |  | 0.36 ±0.30 | 0.33 ±0.23 | 0.621 |
| #: Fisher’s exact test was used; ARB= angiotensin receptor blockers; ACEI= angiotensin converting enzyme inhibitors; LVEF= left ventricular ejection fraction; T-ch= serum cholesterol; BUN= serum urea nitrogen; Hb= hemoglobin; PLt= Platelet count; INR= international normalized ratio. | | | | | |

| **S1 Table L. Operative Characteristics between patients with resternotomy for postoperative bleeding or not.** | | | | |
| --- | --- | --- | --- | --- |
| Characteristics |  | Resternotomy for postoperative bleeding | | p-value |
|  |  | Yes (N= 26) | No (N= 1982) |  |
| Redo surgery |  | 2(7.7%) | 26(1.3%) | 0.050# |
| Emergent operation |  | 3(11.5%) | 16(0.8%) | 0.002# |
| **Type of surgery, no. (%)** |  |  |  | 0.012 |
| CABG Only |  | 2(7.7%) | 212(10.7%) |  |
| Aortic valve |  | 3(11.5%) | 238(12.0%) |  |
| Mitral valve |  | 5(19.2%) | 363(18.3%) |  |
| Tricuspid valve |  | 0(0%) | 98(4.9%) |  |
| Complex cardiac |  | 13(50.0%) | 724(36.5%) |  |
| Transplant |  | 1(3.8%) | 5(0.3%) |  |
| Aortic |  | 2(7.7%) | 83(4.2%) |  |
| Others |  | 0(0%) | 259(13.1%) |  |
| Duration of anesthesia [mean(SD); min] |  | 363 ± 182 | 309 ± 104 | 0.144 |
| Duration of surgery [mean(SD); min] |  | 281 ± 136 | 264 ± 101 | 0.390 |
| CPB time [mean(SD); min] |  | 144 ± 64 | 128 ± 61 | 0.188 |
| IABA, no. (%) |  | 0(0%) | 10(0.5%) | 1# |
| | #: Fisher’s exact test was used; Redo surgery= ≥1 previous sternotomy; CABG= coronary artery bypass grafting; Aortic valve surgery= without ascending aortic replacement; Complex cardiac surgery= combined coronary artery bypass graft surgery and valve surgery or multi-valve surgery; Aortic surgery= Aortic dissections, type A and B, thoracic aortic aneurysms) or Aortic valve surgery with ascending aortic replacement; Others surgery type including ASD (atrial septal defect), VSD (interventricular septal defect), LAM (atrial myxoma), ASV (Aneurysm Sinus Valsalva), CPF (coronary artery pulmonary artery fistula), patent foramen ovale (PFO)/atrial septal aneurysm surgery, and surgery for cardiac tumors; IABA= intra-aortic balloon pump. | | --- | | | | | |

| **Continue S1 Table L. Operative Characteristics between patients with resternotomy for postoperative bleeding or not.** | | | | |
| --- | --- | --- | --- | --- |
| Characteristics |  | Resternotomy for postoperative bleeding | | p-value |
|  |  | Yes (N= 26) | No (N= 1982) |  |
| **Intra-operative transfusion** |  |  |  |  |
| RBCs transfusion, no. (%) |  | 10(38.5%) | 453(22.9%) | 0.061 |
| FFP transfusion, no. (%) |  | 19(73.1%) | 717(36.2%) | < 0.001 |
| Platelet transfusion, no. (%) |  | 2(7.7%) | 178(9.0%) | 1# |
| **Input and Output Characteristics** |  |  |  |  |
| Blood loss [mean(SD); mL] |  | 788 ± 379 | 674 ± 390 | 0.147 |
| Urine output [mean(SD); mL] |  | 919 ± 530 | 764 ± 489 | 0.107 |
| Pump blood [mean(SD); mL] |  | 538 ± 117 | 515 ± 133 | 0.375 |
| Autologous transfusion [median(IQR); mL] |  | 463(250~664) | 300(200~500) | 0.137 |
| ANH [median(IQR); mL] |  | 0(0~200) | 0(0~0) | 0.616 |
| Introperative Crystals [mean(SD); mL] |  | 1615 ± 816 | 1510 ± 570 | 0.519 |
| Introperative Colloid [mean(SD); mL] |  | 769 ± 430 | 685 ± 374 | 0.256 |
| **Intra-operative coagulation drugs** |  |  |  |  |
| PCC, no. (%) |  | 1(3.8%) | 57(2.9%) | 0.536# |
| Fb, no. (%) |  | 1(3.8%) | 65(3.3%) | 0.583# |
| rFVIIa, no. (%) |  | 2(7.7%) | 6(0.3%) | 0.004# |
| TXA, no. (%) |  | 7(26.9%) | 656(33.1%) | 0.506 |
| | #: Fisher’s exact test was used; RBCs= red blood cells; PCC= Prothrombin Complex Concentrate; Fb= Fibrinogen concentrate; rFVIIa= recombinant activated factor VII; Hb= hemoglobin; Hct= hematocrit; TXA= Tranexamic acid. | | --- | | | | | |
