# Supplementary material for: Intraoperative tranexamic acid is associated with postoperative stroke in patients undergoing cardiac surgery
Source: PLoS One. 2017 May 26;12(5):e0177011. doi: 10.1371/journal.pone.0177011 (PMC5446127; doi:10.1371/journal.pone.0177011)
Supplement: S2 Table — (DOC) [file pone.0177011.s002.doc]

| **S2 Table A. Postoperative Complications after excluding the patients undergoing CABG surgeries.** | | | | |
| --- | --- | --- | --- | --- |
| **Outcomes** | Entire Sample | | Adjusted OR | *p*-value |
|  | TXA | Non-TXA |  |  |
| **Stroke** |  |  |  |  |
| no | 573(98.5%) | 1134(99.6%) | Ref |  |
| yes | 9(1.5%) | 4(0.4%) | 4.39 (1.19~16.19) | 0.026 |
| **Seizure** |  |  |  |  |
| no | 576(99.0%) | 1127(99.0%) | Ref |  |
| yes | 6(1.0%) | 11(1.0%) | 1.30 (0.40~4.19) | 0.665 |
| **Coma** |  |  |  |  |
| no | 581(99.8%) | 1131(99.4%) | Ref |  |
| yes | 1(0.2%) | 7(0.6%) | 1.25 (0.10~16.23) | 0.866 |
| **Death** |  |  |  |  |
| no | 573(98.5%) | 1122(98.6%) | Ref |  |
| yes | 9(1.5%) | 16(1.4%) | 1.28 (0.47~3.45) | 0.625 |
| **CRRT** |  |  |  |  |
| no | 569(97.8%) | 1124(98.8%) | Ref |  |
| yes | 13(2.2%) | 14(1.2%) | 1.10 (0.43~2.84) | 0.836 |
| **Resternotomy for postoperative bleeding** | | | | |
| no | 576(99.0%) | 1121(98.5%) | Ref |  |
| yes | 6(1.0%) | 17(1.5%) | 1.70 (0.55~5.25) | 0.356 |
| CRRT= continuous renal replacement therapy; TXA= Tranexamic acid. | | | | |

| **S2 Table B. Ventilation time, ICU and LOS Stay after excluding the patients undergoing CABG surgeries.** | | | | |
| --- | --- | --- | --- | --- |
|  | TXA | | Adjusted B (SE) | *p-* valve |
|  | Yes (n= 574) | No (n= 1125) |  |  |
| Ventilation [median(IQR); hours] | 16(8~20) | 14(8~20) | -0.78 (2.44) | 0.750 |
| ICU [median(IQR); days] | 4(3~6) | 4(2~5) | -0.14 (0.26) | 0.591 |
| LOS [median(IQR); days] | 12(9~15) | 10(7~13) | 0.24 (0.58) | 0.685 |
| ICU= intensive care unit; LOS= length of hospital stay; TXA= Tranexamic acid. | | | | |
