# Supplementary material for: Intraoperative tranexamic acid is associated with postoperative stroke in patients undergoing cardiac surgery
Source: PLoS One. 2017 May 26;12(5):e0177011. doi: 10.1371/journal.pone.0177011 (PMC5446127; doi:10.1371/journal.pone.0177011)
Supplement: S3 Table — (DOC) [file pone.0177011.s003.doc]

| **S3 Table A.** **Demographic and Clinical characteristics between patients with stroke or not after excluding the patients undergoing CABG surgery.** | | | | | |
| --- | --- | --- | --- | --- | --- |
| Preoperative Characteristics |  | Stroke | | p-value | |
|  |  | Yes (N= 13) | No (N= 1707) |  |  |
| Age [mean(SD); yr] |  | 58 ± 11 | 50 ± 13 | 0.039 | |
| Male/female, no. (%) |  | 7/6(53.8%) | 742/965(43.5%) | 0.452 | |
| BMI [mean(SD); kg/m^2^] |  | 21.5 ± 4.3 | 22.1 ± 3.1 | 0.610 | |
| ASA, no. (%) |  |  |  | 0.820 | |
| I |  | 0(0%) | 6(0.4%) |  | |
| II |  | 3(23.1%) | 361(21.1%) |  | |
| III |  | 8(61.5%) | 1216(71.2%) |  | |
| IV |  | 2(15.4%) | 120(7.0%) |  | |
| V |  | 0(0%) | 4(0.2%) |  | |
| NYHA class III/IV, no.(%) |  | 7(53.8%) | 507(29.7%) | 0.058 | |
| History of smoking, no. (%) |  | 5(38.5%) | 346(20.3%) | 0.105 | |
| **Coexistent disease** | | | | | |
| AF, no. (%) |  | 4(30.8%) | 464(27.2%) | 0.759# | |
| Hypertension, no. (%) |  | 3(23.1%) | 285(16.7%) | 0.466# | |
| Diabetes, no. (%) |  | 0(0%) | 63(3.7%) | 1# | |
| HLP, no. (%) |  | 0(0%) | 5(0.3%) | 1# | |
| Cerebrovascular disease, no. (%) |  | 2(15.4%) | 91(5.3%) | 0.153# | |
| CKD, no. (%) |  | 0(0%) | 21(1.2%) | 1# | |
| Liver dysfunction, no. (%) |  | 0(0%) | 11(0.6%) | 1# | |
| COPD, no. (%) |  | 0(0%) | 22(1.3%) | 1# | |
| Infective Endocarditis, no. (%) |  | 0(0%) | 39(2.3%) | 1# | |
| MI, no. (%) |  | 0(0%) | 7(0.4%) | 1# | |
| Preoperative shock, no. (%) |  | 0(0%) | 1(0.1%) | 1# | |
| Preoperative anemia, no. (%) |  | 1(7.7%) | 211(12.6%) | 1# | |
| #: Fisher’s exact test was used; BMI= body mass index; ASA= American Society of Anesthesiologists; NYHA= New York Heart Association; AF= atrial fibrillation; HLP= Hyperlipidaemia; CKD= chronic kidney disease; COPD= chronic obstructive pulmonary disease; MI= myocardial infarction in 30 days before operation. | | | | |  |

| **Continue S3 Table A. Demographic and Clinical characteristics between patients with stroke or not.** | | | | |
| --- | --- | --- | --- | --- |
| Preoperative Characteristics |  | Stroke | | p-value |
|  |  | Yes (N= 13) | No (N= 1707) |  |
| **Preoperative Medication** | | | | |
| ARB or ACEI, no. (%) |  | 0(0%) | 97(5.7%) | 1# |
| β-blockers, no.(%) |  | 0(0%) | 105(6.2%) | 1# |
| Calcium Channel Blockers, no. (%) |  | 0(0%) | 77(4.5%) | 1# |
| Nitrates, no. (%) |  | 0(0%) | 45(2.6%) | 1# |
| Coumadin, no. (%) |  | 1(7.7%) | 78(4.6%) | 0.459# |
| Heparin, no. (%) |  | 0(0%) | 2(0.1%) | 1# |
| Clopidogrel, no. (%) |  | 0(0%) | 12(0.7%) | 1# |
| Aspirin, no. (%) |  | 1(7.7%) | 93(5.4%) | 0.520# |
| Statin use, no.(%) |  | 0(0%) | 33(1.9%) | 1# |
| Diuretics, no. (%) |  | 3(23.1%) | 147(8.6%) | 0.097# |
| Digoxin, no. (%) |  | 1(7.7%) | 100(5.9%) | 0.546# |
| **Preoperative laboratory examination** | | | | |
| LVEF <35%, no.(%) |  | 1(7.7%) | 8(0.5%) | 0.066# |
| T-ch [mean(SD); mmol/L] |  | 4.0 ± 0.7 | 4.3 ± 1.0 | 0.198 |
| BUN [mean(SD); mmol/L] |  | 6.6 ± 1.9 | 5.9 ± 2.1 | 0.302 |
| Hb [mean(SD); g ·L^-1^] |  | 137 ± 15 | 132 ± 19 | 0.357 |
| PLt [mean(SD); 10^3^/mm^3^] |  | 141 ± 53 | 169 ± 57 | 0.086 |
| INR [mean(SD); seconds] |  | 1.06 ± 0.07 | 1.09 ± 0.29 | 0.650 |
| Propensity score [median(SD)] |  | 0.43 ±0.28 | 0.34 ±0.23 | 0.148 |
| #: Fisher’s exact test was used; ARB= angiotensin receptor blockers; ACEI= angiotensin converting enzyme inhibitors; LVEF= left ventricular ejection fraction; T-ch= serum cholesterol; BUN= serum urea nitrogen; Hb= hemoglobin; PLt= Platelet count; INR= international normalized ratio. | | | | |

| **S3 Table B.** **Operative Characteristics between patients with stroke or not** | | | | |
| --- | --- | --- | --- | --- |
| Characteristics |  | Stroke | | p-value |
|  |  | Yes (N= 13) | No (N= 1707) |  |
| Redo surgery |  | 1(7.7%) | 26(1.5%) | 0.187# |
| Emergent operation |  | 1(7.7%) | 13(0.8%) | 0.101# |
| **Type of surgery, no. (%)** |  |  |  | 0.750 |
| Aortic valve |  | 0(0%) | 241(14.1%) |  |
| Mitral valve |  | 3(23.1%) | 365(21.4%) |  |
| Tricuspid valve |  | 1(7.7%) | 97(5.7%) |  |
| Complex cardiac |  | 7(53.8%) | 656(38.4%) |  |
| Transplant |  | 0(0%) | 6(0.4%) |  |
| Aortic |  | 1(7.7%) | 84(4.9%) |  |
| Others |  | 1(7.7%) | 258(15.1%) |  |
| Duration of anesthesia [mean(SD); min] |  | 390 ± 235 | 290 ± 92 | 0.154 |
| Duration of surgery [mean(SD); min] |  | 343 ± 238 | 246 ± 89 | 0.166 |
| CPB time [mean(SD); min] |  | 172 ± 157 | 122 ± 57 | 0.272 |
| IABA, no. (%) |  | 0(0%) | 6(0.4%) | 1# |
| **Intra-operative transfusion** |  |  |  |  |
| RBCs transfusion, no. (%) |  | 3(23.1%) | 332(19.4%) | 0.726# |
| FFP transfusion, no. (%) |  | 6(46.2%) | 579(33.9%) | 0.354 |
| Platelet transfusion, no. (%) |  | 0(0%) | 80(4.7%) | 1# |
| **Input and Output Characteristics** |  |  |  |  |
| Blood loss [mean(SD); mL] |  | 1146 ± 1238 | 657 ± 380 | 0.180 |
| Urine output [mean(SD); mL] |  | 677 ± 306 | 711 ± 419 | 0.768 |
| Pump blood [mean(SD); mL] |  | 462 ± 77 | 510 ± 127 | 0.167 |
| Autologous transfusion [median(IQR); mL] |  | 470(250~500) | 300(200~500) | 0.355 |
| ANH [median(IQR); mL] |  | 0(0~200) | 0(0~0) | 0.671 |
| Introperative Crystals [mean(SD); mL] |  | 1423 ± 400 | 1469 ± 553 | 0.765 |
| Introperative Colloid [mean(SD); mL] |  | 885 ± 506 | 680 ± 364 | 0.171 |
| **Intra-operative coagulation drugs** |  |  |  |  |
| Prothrombin Complex Concentrate, no. (%) |  | 0(0%) | 45(2.6%) | 1# |
| Fibrinogen concentrate, no. (%) |  | 0(0%) | 54(3.2%) | 1# |
| recombinant activated factor VII, no. (%) |  | 0(0%) | 6(0.4%) | 1# |
| Tranexamic acid, no. (%) |  | 9(69.2%) | 573(33.6%) | 0.007 |
| | #: Fisher’s exact test was used; Redo surgery= ≥1 previous sternotomy; Aortic valve surgery= without ascending aortic replacement; Complex cardiac surgery= multi-valve surgery; Aortic surgery= Aortic dissections, type A and B, thoracic aortic aneurysms) or Aortic valve surgery with ascending aortic replacement; Others surgery type including ASD (atrial septal defect), VSD (interventricular septal defect), LAM (atrial myxoma), ASV (Aneurysm Sinus Valsalva), CPF (coronary artery pulmonary artery fistula), patent foramen ovale (PFO)/atrial septal aneurysm surgery, and surgery for cardiac tumors; CPB= cardiopulmonary bypass; IABA= intra-aortic balloon pump; RBCs= red blood cells. | | --- | | | | | |

| **S3 Table C. Demographic and Clinical characteristics between patients with seizure or not.** | | | | | |
| --- | --- | --- | --- | --- | --- |
| Preoperative Characteristics |  | Seizure | | p-value | |
|  |  | Yes (N= 17) | No (N= 1703) |  |  |
| Age [mean(SD); yr] |  | 49 ± 17 | 50 ± 13 | 0.674 | |
| Male/female, no. (%) |  | 8/9(47.1%) | 741/962(43.5%) | 0.769 | |
| BMI [mean(SD); kg/m^2^] |  | 21.3 ± 3.4 | 22.1 ± 3.1 | 0.322 | |
| ASA, no. (%) |  |  |  | 0.603 | |
| I |  | 0(0%) | 6(0.4%) |  | |
| II |  | 2(11.8%) | 362(21.3%) |  | |
| III |  | 15(88.2%) | 1209(71.0%) |  | |
| IV |  | 0(0%) | 122(7.2%) |  | |
| V |  | 0(0%) | 4(0.2%) |  | |
| NYHA class III/IV, no.(%) |  | 5(29.4%) | 509(29.9%) | 0.966 | |
| History of smoking, no. (%) |  | 5(29.4%) | 346(20.3%) | 0.355 | |
| **Coexistent disease** | | | | | |
| AF, no. (%) |  | 4(23.5%) | 464(27.2%) | 1# | |
| Hypertension, no. (%) |  | 7(41.2%) | 281(16.5%) | 0.007 | |
| Diabetes, no. (%) |  | 1(5.9%) | 62(3.6%) | 0.471# | |
| HLP, no. (%) |  | 0(0%) | 5(0.3%) | 1# | |
| Cerebrovascular disease, no. (%) |  | 1(5.9%) | 92(5.4%) | 0.613# | |
| CKD, no. (%) |  | 1(5.9%) | 20(1.2%) | 0.189# | |
| Liver dysfunction, no. (%) |  | 0(0%) | 11(0.6%) | 1# | |
| COPD, no. (%) |  | 0(0%) | 22(1.3%) | 1# | |
| Infective Endocarditis, no. (%) |  | 0(0%) | 39(2.3%) | 1# | |
| MI, no. (%) |  | 0(0%) | 7(0.4%) | 1# | |
| Preoperative shock, no. (%) |  | 0(0%) | 1(0.1%) | 1# | |
| Preoperative anemia, no. (%) |  | 2(11.8%) | 210(12.5%) | 1# | |
| #: Fisher’s exact test was used; BMI= body mass index; ASA= American Society of Anesthesiologists; NYHA= New York Heart Association; AF= atrial fibrillation; HLP= Hyperlipidaemia; CKD= chronic kidney disease; COPD= chronic obstructive pulmonary disease; MI= myocardial infarction in 30 days before operation. | | | | |  |

| **Continue S3 Table C. Demographic and Clinical characteristics between patients with seizure or not.** | | | | |
| --- | --- | --- | --- | --- |
| Preoperative Characteristics |  | Seizure | | p-value |
|  |  | Yes (N= 17) | No (N= 1703) |  |
| **Preoperative Medication** | | | | |
| ARB or ACEI, no. (%) |  | 2(11.8%) | 95(5.6%) | 0.248# |
| β-blockers, no.(%) |  | 3(17.6%) | 102(6.0%) | 0.080# |
| Calcium Channel Blockers, no. (%) |  | 1(5.9%) | 76(4.5%) | 0.543# |
| Nitrates, no. (%) |  | 0(0%) | 45(2.6%) | 1# |
| Coumadin, no. (%) |  | 0(0%) | 79(4.6%) | 1# |
| Heparin, no. (%) |  | 0(0%) | 2(0.1%) | 1# |
| Clopidogrel, no. (%) |  | 1(5.9%) | 11(0.6%) | 0.113# |
| Aspirin, no. (%) |  | 1(5.9%) | 93(5.5%) | 0.617# |
| Statin use, no.(%) |  | 0(0%) | 33(1.9%) | 1# |
| Diuretics, no. (%) |  | 1(5.9%) | 149(8.7%) | 1# |
| Digoxin, no. (%) |  | 0(0%) | 101(5.9%) | 0.619# |
| **Preoperative laboratory examination** | | | | |
| LVEF <35%, no.(%) |  | 1(5.9%) | 8(0.5%) | 0.086# |
| T-ch [mean(SD); mmol/L] |  | 4.5 ± 0.9 | 4.3 ± 1.0 | 0.379 |
| BUN [mean(SD); mmol/L] |  | 6.7 ± 2.7 | 5.9 ± 2.1 | 0.144 |
| Hb [mean(SD); g ·L^-1^] |  | 136 ± 23 | 132 ± 19 | 0.427 |
| PLt [mean(SD); 10^3^/mm^3^] |  | 159 ± 40 | 169 ± 58 | 0.505 |
| INR [mean(SD); seconds] |  | 1.06 ± 0.12 | 1.09 ± 0.29 | 0.675 |
| Propensity score [median(SD)] |  | 0.41 ±0.26 | 0.34 ±0.23 | 0.203 |
| #: Fisher’s exact test was used; ARB= angiotensin receptor blockers; ACEI= angiotensin converting enzyme inhibitors; LVEF= left ventricular ejection fraction; T-ch= serum cholesterol; BUN= serum urea nitrogen; Hb= hemoglobin; PLt= Platelet count; INR= international normalized ratio. | | | | |

| **S3 Table D. Operative Characteristics between patients with seizure or not** | | | | |
| --- | --- | --- | --- | --- |
| Characteristics |  | Seizure | | p-value |
|  |  | Yes (N= 17) | No (N= 1703) |  |
| Redo surgery |  | 0(0%) | 27(1.6%) | 1# |
| Emergent operation |  | 0(0%) | 14(0.8%) | 1# |
| **Type of surgery, no. (%)** |  |  |  | 0.068 |
| Aortic valve |  | 2(11.8%) | 239(14.0%) |  |
| Mitral valve |  | 9(52.9%) | 359(21.1%) |  |
| Tricuspid valve |  | 1(5.9%) | 97(5.7%) |  |
| Complex cardiac |  | 4(23.5%) | 659(38.7%) |  |
| Transplant |  | 0(0%) | 6(0.4%) |  |
| Aortic |  | 1(5.9%) | 84(4.9%) |  |
| Others |  | 0(0%) | 259(15.2%) |  |
| Duration of anesthesia [mean(SD); min] |  | 339 ± 116 | 291 ± 93 | 0.034 |
| Duration of surgery [mean(SD); min] |  | 294 ± 112 | 246 ± 91 | 0.031 |
| CPB time [mean(SD); min] |  | 141 ± 66 | 122 ± 59 | 0.181 |
| IABA, no. (%) |  | 0(0%) | 6(0.4%) | 1# |
| **Intra-operative transfusion** |  |  |  |  |
| RBCs transfusion, no. (%) |  | 3(17.6%) | 332(19.5%) | 1# |
| FFP transfusion, no. (%) |  | 6(35.3%) | 579(34.0%) | 0.911 |
| Platelet transfusion, no. (%) |  | 2(11.8%) | 78(4.6%) | 0.186# |
| **Input and Output Characteristics** |  |  |  |  |
| Blood loss [mean(SD); mL] |  | 741 ± 402 | 660 ± 395 | 0.401 |
| Urine output [mean(SD); mL] |  | 881 ± 548 | 709 ± 417 | 0.091 |
| Pump blood [mean(SD); mL] |  | 568 ± 140 | 509 ± 127 | 0.060 |
| Autologous transfusion [median(IQR); mL] |  | 350(250~475) | 300(200~500) | 0.709 |
| ANH [median(IQR); mL] |  | 0(0~0) | 0(0~0) | 0.075 |
| Introperative Crystals [mean(SD); mL] |  | 1382 ± 600 | 1470 ± 552 | 0.517 |
| Introperative Colloid [mean(SD); mL] |  | 735 ± 400 | 681 ± 365 | 0.540 |
| **Intra-operative coagulation drugs** |  |  |  |  |
| Prothrombin Complex Concentrate, no. (%) |  | 0(0%) | 45(2.6%) | 1# |
| Fibrinogen concentrate, no. (%) |  | 0(0%) | 54(3.2%) | 1# |
| recombinant activated factor VII, no. (%) |  | 0(0%) | 6(0.4%) | 1# |
| Tranexamic acid, no. (%) |  | 6(35.3%) | 576(33.8%) | 0.898 |
| Propensity score [median(SD)] |  | 0.41 ±0.26 | 0.34 ±0.23 | 0.203 |
| | #: Fisher’s exact test was used; Redo surgery= ≥1 previous sternotomy; Aortic valve surgery= without ascending aortic replacement; Complex cardiac surgery= multi-valve surgery; Aortic surgery= Aortic dissections, type A and B, thoracic aortic aneurysms) or Aortic valve surgery with ascending aortic replacement; Others surgery type including ASD (atrial septal defect), VSD (interventricular septal defect), LAM (atrial myxoma), ASV (Aneurysm Sinus Valsalva), CPF (coronary artery pulmonary artery fistula), patent foramen ovale (PFO)/atrial septal aneurysm surgery, and surgery for cardiac tumors; CPB= cardiopulmonary bypass; IABA= intra-aortic balloon pump; RBCs= red blood cells. |  | #: Fisher’s exact test was used; Redo surgery= ≥1 previous sternotomy; Aortic valve surgery= without ascending aortic replacement; Complex cardiac surgery= multi-valve surgery; Aortic surgery= Aortic dissections, type A and B, thoracic aortic aneurysms) or Aortic valve surgery with ascending aortic replacement; Others surgery type including ASD (atrial septal defect), VSD (interventricular septal defect), LAM (atrial myxoma), ASV (Aneurysm Sinus Valsalva), CPF (coronary artery pulmonary artery fistula), patent foramen ovale (PFO)/atrial septal aneurysm surgery, and surgery for cardiac tumors; CPB= cardiopulmonary bypass; IABA= intra-aortic balloon pump; RBCs= red blood cells. | | --- | --- | --- | | | | | |

| **S3 Table E. Demographic and Clinical characteristics between patients with coma or not.** | | | | | |
| --- | --- | --- | --- | --- | --- |
| Preoperative Characteristics |  | Coma | | p-value | |
|  |  | Yes (N= 8) | No (N= 1712) |  |  |
| Age [mean(SD); yr] |  | 49 ± 14 | 50 ± 13 | 0.713 | |
| Male/female, no. (%) |  | 6/2(75.0%) | 743/969(43.4%) | 0.085# | |
| BMI [mean(SD); kg/m^2^] |  | 22.3 ± 3.6 | 22.1 ± 3.1 | 0.805 | |
| ASA, no. (%) |  |  |  | 0.399 | |
| I |  | 0(0%) | 6(0.4%) |  | |
| II |  | 1(12.5%) | 363(21.2%) |  | |
| III |  | 5(62.5%) | 1219(71.2%) |  | |
| IV |  | 2(25.0%) | 120(7.0%) |  | |
| V |  | 0(0%) | 4(0.2%) |  | |
| NYHA class III/IV, no.(%) |  | 4(50.0%) | 510(29.8%) | 0.250# | |
| History of smoking, no. (%) |  | 3(37.5%) | 348(20.3%) | 0.212# | |
| **Coexistent disease** | | | | | |
| AF, no. (%) |  | 0(0%) | 468(27.3%) | 0.117# | |
| Hypertension, no. (%) |  | 3(37.5%) | 285(16.6%) | 0.136# | |
| Diabetes, no. (%) |  | 0(0%) | 63(3.7%) | 1# | |
| HLP, no. (%) |  | 0(0%) | 5(0.3%) | 1# | |
| Cerebrovascular disease, no. (%) |  | 1(12.5%) | 92(5.4%) | 0.360# | |
| CKD, no. (%) |  | 0(0%) | 21(1.2%) | 1# | |
| Liver dysfunction, no. (%) |  | 1(12.5%) | 10(0.6%) | 0.050# | |
| COPD, no. (%) |  | 0(0%) | 22(1.3%) | 1# | |
| Infective Endocarditis, no. (%) |  | 0(0%) | 39(2.3%) | 1# | |
| MI, no. (%) |  | 0(0%) | 7(0.4%) | 1# | |
| Preoperative shock, no. (%) |  | 0(0%) | 1(0.1%) | 1# | |
| Preoperative anemia, no. (%) |  | 1(12.5%) | 211(12.5%) | 1# | |
| #: Fisher’s exact test was used; BMI= body mass index; ASA= American Society of Anesthesiologists; NYHA= New York Heart Association; AF= atrial fibrillation; HLP= Hyperlipidaemia; CKD= chronic kidney disease; COPD= chronic obstructive pulmonary disease; MI= myocardial infarction in 30 days before operation. | | | | |  |

| **Continue S3 Table E. Demographic and Clinical characteristics between patients with coma or not.** | | | | |
| --- | --- | --- | --- | --- |
| Preoperative Characteristics |  | Coma | | p-value |
|  |  | Yes (N= 8) | No (N= 1712) |  |
| **Preoperative Medication** | | | | |
| ARB or ACEI, no. (%) |  | 0(0%) | 97(5.7%) | 1# |
| β-blockers, no.(%) |  | 0(0%) | 105(6.1%) | 1# |
| Calcium Channel Blockers, no. (%) |  | 0(0%) | 77(4.5%) | 1# |
| Nitrates, no. (%) |  | 0(0%) | 45(2.6%) | 1# |
| Coumadin, no. (%) |  | 1(12.5%) | 78(4.6%) | 0.314# |
| Heparin, no. (%) |  | 0(0%) | 2(0.1%) | 1# |
| Clopidogrel, no. (%) |  | 0(0%) | 12(0.7%) | 1# |
| Aspirin, no. (%) |  | 0(0%) | 94(5.5%) | 1# |
| Statin use, no.(%) |  | 1(12.5%) | 32(1.9%) | 0.144# |
| Diuretics, no. (%) |  | 2(25.0%) | 148(8.6%) | 0.149# |
| Digoxin, no. (%) |  | 0(0%) | 101(5.9%) | 1# |
| **Preoperative laboratory examination** | | | | |
| LVEF <35%, no.(%) |  | 0(0%) | 9(0.5%) | 1# |
| T-ch [mean(SD); mmol/L] |  | 3.9 ± 0.8 | 4.3 ± 1.0 | 0.230 |
| BUN [mean(SD); mmol/L] |  | 5.8 ± 1.0 | 6.0 ± 2.1 | 0.794 |
| Hb [mean(SD); g ·L^-1^] |  | 139 ± 17 | 132 ± 19 | 0.315 |
| PLt [mean(SD); 10^3^/mm^3^] |  | 118 ± 50 | 169 ± 57 | 0.013 |
| INR [mean(SD); seconds] |  | 1.15 ± 0.16 | 1.09 ± 0.29 | 0.601 |
| Propensity score [median(SD)] |  | 0.32 ±0.24 | 0.34 ±0.23 | 0.791 |
| #: Fisher’s exact test was used; ARB= angiotensin receptor blockers; ACEI= angiotensin converting enzyme inhibitors; LVEF= left ventricular ejection fraction; T-ch= serum cholesterol; BUN= serum urea nitrogen; Hb= hemoglobin; PLt= Platelet count; INR= international normalized ratio. | | | | |

| **S3 Table F. Operative Characteristics between patients with coma or not** | | | | |
| --- | --- | --- | --- | --- |
| Characteristics |  | Coma | | p-value |
|  |  | Yes (N= 8) | No (N= 1712) |  |
| Redo surgery |  | 0(0%) | 27(1.6%) | 1# |
| Emergent operation |  | 2(25.0%) | 12(0.7%) | 0.002# |
| **Type of surgery, no. (%)** |  |  |  | < 0.001 |
| Aortic valve |  | 1(12.5%) | 240(14.0%) |  |
| Mitral valve |  | 0(0%) | 368(21.5%) |  |
| Tricuspid valve |  | 0(0%) | 98(5.7%) |  |
| Complex cardiac |  | 2(25.0%) | 661(38.6%) |  |
| Transplant |  | 0(0%) | 6(0.4%) |  |
| Aortic |  | 4(50.0%) | 81(4.7%) |  |
| Others |  | 1(12.5%) | 258(15.1%) |  |
| Duration of anesthesia [mean(SD); min] |  | 509 ± 215 | 290 ± 92 | 0.024 |
| Duration of surgery [mean(SD); min] |  | 462 ± 201 | 246 ± 89 | 0.019 |
| CPB time [mean(SD); min] |  | 249 ± 95 | 121 ± 58 | 0.007 |
| IABA, no. (%) |  | 0(0%) | 6(0.4%) | 1# |
| **Intra-operative transfusion** |  |  |  |  |
| RBCs transfusion, no. (%) |  | 5(62.5%) | 330(19.3%) | 0.009# |
| FFP transfusion, no. (%) |  | 6(75.0%) | 579(33.8%) | 0.022# |
| Platelet transfusion, no. (%) |  | 1(12.5%) | 79(4.6%) | 0.317# |
| **Input and Output Characteristics** |  |  |  |  |
| Blood loss [mean(SD); mL] |  | 1513 ± 1667 | 657 ± 377 | 0.190 |
| Urine output [mean(SD); mL] |  | 850 ± 600 | 710 ± 417 | 0.346 |
| Pump blood [mean(SD); mL] |  | 500 ± 93 | 510 ± 127 | 0.823 |
| Autologous transfusion [median(IQR); mL] |  | 500(300~1368) | 300(200~500) | 0.064 |
| ANH [median(IQR); mL] |  | 0(0~150) | 0(0~0) | 0.991 |
| Introperative Crystals [mean(SD); mL] |  | 1750 ± 378 | 1467 ± 553 | 0.149 |
| Introperative Colloid [mean(SD); mL] |  | 650 ± 407 | 681 ± 366 | 0.809 |
| **Intra-operative coagulation drugs** |  |  |  |  |
| Prothrombin Complex Concentrate, no. (%) |  | 1(12.5%) | 44(2.6%) | 0.191# |
| Fibrinogen concentrate, no. (%) |  | 1(12.5%) | 53(3.1%) | 0.226# |
| recombinant activated factor VII, no. (%) |  | 1(12.5%) | 5(0.3%) | 0.028# |
| Tranexamic acid, no. (%) |  | 1(12.5%) | 581(33.9%) | 0.279# |
| | #: Fisher’s exact test was used; Redo surgery= ≥1 previous sternotomy; Aortic valve surgery= without ascending aortic replacement; Complex cardiac surgery= multi-valve surgery; Aortic surgery= Aortic dissections, type A and B, thoracic aortic aneurysms) or Aortic valve surgery with ascending aortic replacement; Others surgery type including ASD (atrial septal defect), VSD (interventricular septal defect), LAM (atrial myxoma), ASV (Aneurysm Sinus Valsalva), CPF (coronary artery pulmonary artery fistula), patent foramen ovale (PFO)/atrial septal aneurysm surgery, and surgery for cardiac tumors; CPB= cardiopulmonary bypass; IABA= intra-aortic balloon pump; RBCs= red blood cells. |  | | --- | --- | | | | | |

| **S3 Table G. Demographic and Clinical characteristics between patients with death or not.** | | | | | |
| --- | --- | --- | --- | --- | --- |
| Preoperative Characteristics |  | Death | | p-value | |
|  |  | Yes (N= 25) | No (N= 1695) |  |  |
| Age [mean(SD); yr] |  | 58 ± 15 | 50 ± 13 | 0.002 | |
| Male/female, no. (%) |  | 8/17(32.0%) | 741/954(43.7%) | 0.241 | |
| BMI [mean(SD); kg/m^2^] |  | 20.8 ± 3.2 | 22.1 ± 3.1 | 0.043 | |
| ASA, no. (%) |  |  |  | <0.001 | |
| I |  | 0(0%) | 6(0.4%) |  | |
| II |  | 0(0%) | 364(21.5%) |  | |
| III |  | 19(76.0%) | 1205(71.1%) |  | |
| IV |  | 4(16.0%) | 118(7.0%) |  | |
| V |  | 2(8.0%) | 2(0.1%) |  | |
| NYHA class III/IV, no.(%) |  | 13(52.0%) | 501(29.6%) | 0.025 | |
| History of smoking, no. (%) |  | 2(8.0%) | 349(20.6%) | 0.140# | |
| **Coexistent disease** | | | | | |
| AF, no. (%) |  | 10(40.0%) | 458(27.0%) | 0.173 | |
| Hypertension, no. (%) |  | 10(40.0%) | 278(16.4%) | 0.002 | |
| Diabetes, no. (%) |  | 2(8.0%) | 61(3.6%) | 0.232# | |
| HLP, no. (%) |  | 0(0%) | 5(0.3%) | 1# | |
| Cerebrovascular disease, no. (%) |  | 2(8.0%) | 91(5.4%) | 0.396# | |
| CKD, no. (%) |  | 1(4.0%) | 20(1.2%) | 0.266# | |
| Liver dysfunction, no. (%) |  | 0(0%) | 11(0.6%) | 1# | |
| COPD, no. (%) |  | 0(0%) | 22(1.3%) | 1# | |
| Infective Endocarditis, no. (%) |  | 0(0%) | 39(2.3%) | 1# | |
| MI, no. (%) |  | 0(0%) | 7(0.4%) | 1# | |
| Preoperative shock, no. (%) |  | 0(0%) | 1(0.1%) | 1# | |
| Preoperative anemia, no. (%) |  | 9(36.0%) | 203(12.2%) | <0.001 | |
| #: Fisher’s exact test was used; BMI= body mass index; ASA= American Society of Anesthesiologists; NYHA= New York Heart Association; AF= atrial fibrillation; HLP= Hyperlipidaemia; CKD= chronic kidney disease; COPD= chronic obstructive pulmonary disease; MI= myocardial infarction in 30 days before operation. | | | | |  |

| **Continue S3 Table G. Demographic and Clinical characteristics between patients with death or not.** | | | | |
| --- | --- | --- | --- | --- |
| Preoperative Characteristics |  | Death | | p-value |
|  |  | Yes (N= 25) | No (N= 1695) |  |
| **Preoperative Medication** | | | | |
| ARB or ACEI, no. (%) |  | 2(8.0%) | 95(5.6%) | 0.648# |
| β-blockers, no.(%) |  | 3(12.0%) | 102(6.0%) | 0.193# |
| Calcium Channel Blockers, no. (%) |  | 2(8.0%) | 75(4.4%) | 0.309# |
| Nitrates, no. (%) |  | 2(8.0%) | 43(2.5%) | 0.137# |
| Coumadin, no. (%) |  | 0(0%) | 79(4.7%) | 0.626# |
| Heparin, no. (%) |  | 0(0%) | 2(0.1%) | 1# |
| Clopidogrel, no. (%) |  | 0(0%) | 12(0.7%) | 1# |
| Aspirin, no. (%) |  | 3(12.0%) | 91(5.4%) | 0.153# |
| Statin use, no.(%) |  | 2(8.0%) | 31(1.8%) | 0.081# |
| Diuretics, no. (%) |  | 4(16.0%) | 146(8.6%) | 0.268# |
| Digoxin, no. (%) |  | 3(12.0%) | 98(5.8%) | 0.178# |
| **Preoperative laboratory examination** | | | | |
| LVEF <35%, no.(%) |  | 0(0%) | 9(0.5%) | 1# |
| T-ch [mean(SD); mmol/L] |  | 4.3 ± 1.0 | 4.3 ± 1.1 | 0.892 |
| BUN [mean(SD); mmol/L] |  | 7.5 ± 4.2 | 5.9 ± 2.1 | 0.074 |
| Hb [mean(SD); g ·L^-1^] |  | 124 ± 29 | 132 ± 18 | 0.170 |
| PLt [mean(SD); 10^3^/mm^3^] |  | 155 ± 55 | 169 ± 57 | 0.216 |
| INR [mean(SD); seconds] |  | 1.06 ± 0.15 | 1.09 ± 0.29 | 0.588 |
| Propensity score [median(SD)] |  | 0.44 ±0.28 | 0.34 ±0.23 | 0.033 |
| #: Fisher’s exact test was used; ARB= angiotensin receptor blockers; ACEI= angiotensin converting enzyme inhibitors; LVEF= left ventricular ejection fraction; T-ch= serum cholesterol; BUN= serum urea nitrogen; Hb= hemoglobin; PLt= Platelet count; INR= international normalized ratio. | | | | |

| **S3 Table H. Operative Characteristics between patients with death or not.** | | | | |
| --- | --- | --- | --- | --- |
| Characteristics |  | Death | | p-value |
|  |  | Yes (N= 25) | No (N= 1695) |  |
| Redo surgery |  | 2(8.0%) | 25(1.5%) | 0.057# |
| Emergent operation |  | 1(4.0%) | 13(0.8%) | 0.186# |
| **Type of surgery, no. (%)** |  |  |  | 0.097 |
| Aortic valve |  | 2(8.0%) | 239(14.1%) |  |
| Mitral valve |  | 4(16.0%) | 364(21.5%) |  |
| Tricuspid valve |  | 1(4.0%) | 97(5.7%) |  |
| Complex cardiac |  | 13(52.0%) | 650(38.3%) |  |
| Transplant |  | 0(0%) | 6(0.4%) |  |
| Aortic |  | 4(16.0%) | 81(4.8%) |  |
| Others |  | 1(4.0%) | 258(15.2%) |  |
| Duration of anesthesia [mean(SD); min] |  | 435 ± 200 | 289 ± 90 | 0.001 |
| Duration of surgery [mean(SD); min] |  | 380 ± 191 | 245 ± 87 | 0.002 |
| CPB time [mean(SD); min] |  | 173 ± 117 | 121 ± 57 | 0.036 |
| IABA, no. (%) |  | 1(4.0%) | 5(0.3%) | 0.084# |
| **Intra-operative transfusion** |  |  |  |  |
| RBCs transfusion, no. (%) |  | 15(60.0%) | 320(18.9%) | <0.001 |
| FFP transfusion, no. (%) |  | 19(76.0%) | 566(33.4%) | <0.001 |
| Platelet transfusion, no. (%) |  | 7(28.0%) | 73(4.3%) | <0.001 |
| **Input and Output Characteristics** |  |  |  |  |
| Blood loss [mean(SD); mL] |  | 1060 ± 852 | 655 ± 382 | 0.026 |
| Urine output [mean(SD); mL] |  | 760 ± 501 | 710 ± 417 | 0.552 |
| Pump blood [mean(SD); mL] |  | 519 ± 114 | 510 ± 127 | 0.722 |
| Autologous transfusion [median(IQR); mL] |  | 600(250~755) | 300(200~500) | 0.001 |
| ANH [median(IQR); mL] |  | 0(0~0) | 0(0~0) | 0.168 |
| Introperative Crystals [mean(SD); mL] |  | 1660 ± 910 | 1466 ± 545 | 0.298 |
| Introperative Colloid [mean(SD); mL] |  | 900 ± 479 | 678 ± 363 | 0.030 |
| **Intraoperative coagulation drugs** |  |  |  |  |
| Prothrombin Complex Concentrate, no. (%) |  | 1(4.0%) | 44(2.6%) | 0.487# |
| Fibrinogen concentrate, no. (%) |  | 1(4.0%) | 53(3.1%) | 0.552# |
| recombinant activated factor VII, no. (%) |  | 2(8.0%) | 4(0.2%) | 0.003# |
| Tranexamic acid, no. (%) |  | 9(36.0%) | 573(33.8%) | 0.818 |
| | #: Fisher’s exact test was used; Redo surgery= ≥1 previous sternotomy; Aortic valve surgery= without ascending aortic replacement; Complex cardiac surgery= multi-valve surgery; Aortic surgery= Aortic dissections, type A and B, thoracic aortic aneurysms) or Aortic valve surgery with ascending aortic replacement; Others surgery type including ASD (atrial septal defect), VSD (interventricular septal defect), LAM (atrial myxoma), ASV (Aneurysm Sinus Valsalva), CPF (coronary artery pulmonary artery fistula), patent foramen ovale (PFO)/atrial septal aneurysm surgery, and surgery for cardiac tumors; CPB= cardiopulmonary bypass; IABA= intra-aortic balloon pump; RBCs= red blood cells. |  | | --- | --- | | | | | |

| **S3 Table I. Demographic and Clinical characteristics between patients with CRRT or not.** | | | | |
| --- | --- | --- | --- | --- |
| Preoperative Characteristics |  | CRRT | | p-value |
|  |  | Yes (N= 27) | No (N= 1693) |  |
| Age [mean(SD); yr] |  | 59 ± 15 | 50 ± 13 | 0.001 |
| Male/female, no. (%) |  | 12/15(44.4%) | 737/956(43.5%) | 0.924 |
| BMI [mean(SD); kg/m^2^] |  | 21.8 ± 3.5 | 22.1 ± 3.1 | 0.632 |
| ASA, no. (%) |  |  |  | 0.002 |
| I |  | 0(0%) | 6(0.4%) |  |
| II |  | 2(7.4%) | 362(21.4%) |  |
| III |  | 21(77.8%) | 1203(71.1%) |  |
| IV |  | 3(11.1%) | 119(7.0%) |  |
| V |  | 1(3.7%) | 3(0.2%) |  |
| NYHA class III/IV, no.(%) |  | 11(40.7%) | 503(29.7%) | 0.214 |
| History of smoking, no. (%) |  | 6(22.2%) | 345(20.4%) | 0.814 |
| **Coexistent disease** | | | | |
| AF, no. (%) |  | 10(37.0%) | 458(27.1%) | 0.247 |
| Hypertension, no. (%) |  | 13(48.1%) | 275(16.2%) | <0.001 |
| Diabetes, no. (%) |  | 2(7.4%) | 61(3.6%) | 0.260# |
| HLP, no. (%) |  | 0(0%) | 5(0.3%) | 1# |
| Cerebrovascular disease, no. (%) |  | 4(14.8%) | 89(5.3%) | 0.054# |
| CKD, no. (%) |  | 4(14.8%) | 17(1.0%) | <0.001 |
| Liver dysfunction, no. (%) |  | 0(0%) | 11(0.6%) | 1# |
| COPD, no. (%) |  | 0(0%) | 22(1.3%) | 1# |
| Infective Endocarditis, no. (%) |  | 0(0%) | 39(2.3%) | 1# |
| MI, no. (%) |  | 0(0%) | 7(0.4%) | 1# |
| Preoperative shock, no. (%) |  | 0(0%) | 1(0.1%) | 1# |
| Preoperative anemia, no. (%) |  | 9(33.3%) | 203(12.2%) | 0.001 |
| #: Fisher’s exact test was used; BMI= body mass index; ASA= American Society of Anesthesiologists; NYHA= New York Heart Association; AF= atrial fibrillation; HLP= Hyperlipidaemia; CKD= chronic kidney disease; COPD= chronic obstructive pulmonary disease; MI= myocardial infarction in 30 days before operation; CRRT= continuous renal replacement therapy. | | | | |

| **Continue S3 Table I. Demographic and Clinical characteristics between patients with CRRT or not.** | | | | |
| --- | --- | --- | --- | --- |
| Preoperative Characteristics |  | CRRT | | p-value |
|  |  | Yes (N= 27) | No (N= 1693) |  |
| **Preoperative Medication** | | | | |
| ARB or ACEI, no. (%) |  | 3(11.1%) | 94(5.6%) | 0.192# |
| β-blockers, no.(%) |  | 2(7.4%) | 103(6.1%) | 0.679# |
| Calcium Channel Blockers, no. (%) |  | 3(11.1%) | 74(4.4%) | 0.117# |
| Nitrates, no. (%) |  | 1(3.7%) | 44(2.6%) | 0.514# |
| Coumadin, no. (%) |  | 0(0%) | 79(4.7%) | 0.633# |
| Heparin, no. (%) |  | 0(0%) | 2(0.1%) | 1# |
| Clopidogrel, no. (%) |  | 1(3.7%) | 11(0.6%) | 0.173# |
| Aspirin, no. (%) |  | 4(14.8%) | 90(5.3%) | 0.056# |
| Statin use, no.(%) |  | 0(0%) | 33(1.9%) | 1# |
| Diuretics, no. (%) |  | 2(7.4%) | 147(8.7%) | 1# |
| Digoxin, no. (%) |  | 4(14.8%) | 97(5.7%) | 0.069# |
| **Preoperative laboratory examination** | | | | |
| LVEF <35%, no.(%) |  | 1(3.7%) | 8(0.5%) | 0.133# |
| T-ch [mean(SD); mmol/L] |  | 4.3 ± 1.1 | 4.3 ± 1.0 | 0.938 |
| BUN [mean(SD); mmol/L] |  | 7.8 ± 4.0 | 5.9 ± 2.1 | 0.025 |
| Hb [mean(SD); g ·L^-1^] |  | 123 ± 24 | 132 ± 19 | 0.050 |
| PLt [mean(SD); 10^3^/mm^3^] |  | 163 ± 59 | 169 ± 57 | 0.604 |
| INR [mean(SD); seconds] |  | 1.25 ± 0.51 | 1.09 ± 0.28 | 0.126 |
| Propensity score [median(SD)] |  | 0.52 ±0.29 | 0.34 ±0.23 | 0.003 |
| #: Fisher’s exact test was used; ARB= angiotensin receptor blockers; ACEI= angiotensin converting enzyme inhibitors; LVEF= left ventricular ejection fraction; T-ch= serum cholesterol; BUN= serum urea nitrogen; Hb= hemoglobin; PLt= Platelet count; INR= international normalized ratio. | | | | |

| **S3 Table J. Operative Characteristics between patients with CRRT or not.** | | | | |
| --- | --- | --- | --- | --- |
| Characteristics |  | CRRT | | p-value |
|  |  | Yes (N= 27) | No (N= 1693) |  |
| Redo surgery |  | 2(7.4%) | 25(1.5%) | 0.065# |
| Emergent operation |  | 2(7.4%) | 12(0.7%) | 0.019# |
| **Type of surgery, no. (%)** |  |  |  | 0.525 |
| Aortic valve |  | 4(14.8%) | 237(14.0%) |  |
| Mitral valve |  | 7(25.9%) | 361(21.3%) |  |
| Tricuspid valve |  | 0(0%) | 98(5.8%) |  |
| Complex cardiac |  | 11(40.7%) | 652(38.5%) |  |
| Transplant |  | 0(0%) | 6(0.4%) |  |
| Aortic |  | 3(11.1%) | 82(4.8%) |  |
| Others |  | 2(7.4%) | 257(15.2%) |  |
| Duration of anesthesia [mean(SD); min] |  | 421 ± 230 | 289 ± 89 | 0.006 |
| Duration of surgery [mean(SD); min] |  | 342 ± 212 | 245 ± 87 | 0.025 |
| CPB time [mean(SD); min] |  | 177 ± 154 | 121 ± 55 | 0.070 |
| IABA, no. (%) |  | 2(7.4%) | 4(0.2%) | 0.003# |
| **Intra-operative transfusion** |  |  |  |  |
| RBCs transfusion, no. (%) |  | 12(44.4%) | 323(19.1%) | 0.001 |
| FFP transfusion, no. (%) |  | 17(63.0%) | 568(33.5%) | 0.001 |
| Platelet transfusion, no. (%) |  | 5(18.5%) | 75(4.4%) | 0.001 |
| **Input and Output Characteristics** |  |  |  |  |
| Blood loss [mean(SD); mL] |  | 1167 ± 1029 | 653 ± 372 | 0.015 |
| Urine output [mean(SD); mL] |  | 806 ± 673 | 709 ± 413 | 0.463 |
| Pump blood [mean(SD); mL] |  | 508 ± 104 | 510 ± 127 | 0.945 |
| Autologous transfusion [median(IQR); mL] |  | 300(100~600) | 306(200~500) | 0.480 |
| ANH [median(IQR); mL] |  | 0(0~0) | 0(0~0) | 0.322 |
| Introperative Crystals [mean(SD); mL] |  | 1426 ± 661 | 1469 ± 551 | 0.685 |
| Introperative Colloid [mean(SD); mL] |  | 944 ± 577 | 677 ± 360 | 0.024 |
| **Intra-operative coagulation drugs** |  |  |  |  |
| Prothrombin Complex Concentrate, no. (%) |  | 2(7.4%) | 43(2.5%) | 0.156# |
| Fibrinogen concentrate, no. (%) |  | 2(7.4%) | 52(3.1%) | 0.207# |
| recombinant activated factor VII, no. (%) |  | 2(7.4%) | 4(0.2%) | 0.003# |
| Tranexamic acid, no. (%) |  | 13(48.1%) | 569(33.6%) | 0.113 |
| #: Fisher’s exact test was used; Redo surgery= ≥1 previous sternotomy; Aortic valve surgery= without ascending aortic replacement; Complex cardiac surgery= multi-valve surgery; Aortic surgery= Aortic dissections, type A and B, thoracic aortic aneurysms) or Aortic valve surgery with ascending aortic replacement; Others surgery type including ASD (atrial septal defect), VSD (interventricular septal defect), LAM (atrial myxoma), ASV (Aneurysm Sinus Valsalva), CPF (coronary artery pulmonary artery fistula), patent foramen ovale (PFO)/atrial septal aneurysm surgery, and surgery for cardiac tumors; CPB= cardiopulmonary bypass; IABA= intra-aortic balloon pump; RBCs= red blood cells. | | | | |

| **S3 Table K. Demographic and Clinical characteristics between patients with** **resternotomy for postoperative bleeding or not.** | | | | | |
| --- | --- | --- | --- | --- | --- |
| Preoperative Characteristics |  | Resternotomy for  postoperative bleeding | | p-value | |
|  |  | Yes (N= 23) | No (N= 1697) |  |  |
| Age [mean(SD); yr] |  | 57 ± 15 | 50 ± 13 | 0.018 | |
| Male/female, no. (%) |  | 10/13(43.5%) | 739/958(43.5%) | 0.995 | |
| BMI [mean(SD); kg/m^2^] |  | 21.6 ± 3.3 | 22.1 ± 3.1 | 0.485 | |
| ASA, no. (%) |  |  |  | 0.008 | |
| I |  | 0(0%) | 6(0.4%) |  | |
| II |  | 2(8.7%) | 362(21.3%) |  | |
| III |  | 15(65.2%) | 1209(71.2%) |  | |
| IV |  | 6(26.1%) | 116(6.8%) |  | |
| V |  | 0(0%) | 4(0.2%) |  | |
| NYHA class III/IV, no.(%) |  | 12(52.2%) | 502(29.6%) | 0.019 | |
| History of smoking, no. (%) |  | 5(21.7%) | 346(20.4%) | 0.873 | |
| **Coexistent disease** | | | | | |
| AF, no. (%) |  | 10(43.5%) | 458(27.0%) | 0.078 | |
| Hypertension, no. (%) |  | 6(26.1%) | 282(16.6%) | 0.227 | |
| Diabetes, no. (%) |  | 4(17.4%) | 59(3.5%) | 0.009# | |
| HLP, no. (%) |  | 0(0%) | 5(0.3%) | 1# | |
| Cerebrovascular disease, no. (%) |  | 1(4.3%) | 92(5.4%) | 1# | |
| CKD, no. (%) |  | 0(0%) | 21(1.2%) | 1# | |
| Liver dysfunction, no. (%) |  | 0(0%) | 11(0.6%) | 1# | |
| COPD, no. (%) |  | 1(4.3%) | 21(1.2%) | 0.258# | |
| Infective Endocarditis, no. (%) |  | 0(0%) | 39(2.3%) | 1# | |
| MI, no. (%) |  | 1(4.3%) | 6(0.4%) | 0.090# | |
| Preoperative shock, no. (%) |  | 1(4.3%) | 0(0%) | 0.013# | |
| Preoperative anemia, no. (%) |  | 3(13.0%) | 209(12.5%) | 1# | |
| #: Fisher’s exact test was used; BMI= body mass index; ASA= American Society of Anesthesiologists; NYHA= New York Heart Association; AF= atrial fibrillation; HLP= Hyperlipidaemia; CKD= chronic kidney disease; COPD= chronic obstructive pulmonary disease; MI= myocardial infarction in 30 days before operation. | | | | |  |

| **Continue S3 Table K. Demographic and Clinical characteristics between patients with resternotomy for postoperative bleeding or not.** | | | | | |
| --- | --- | --- | --- | --- | --- |
| Preoperative Characteristics |  | | Resternotomy for postoperative bleeding | | p-value |
|  |  | | Yes (N= 23) | No (N= 1697) |  |
| **Preoperative Medication** | | | | | |
| ARB or ACEI, no. (%) | |  | 3(13.0%) | 94(5.5%) | 0.136# |
| β-blockers, no.(%) | |  | 1(4.3%) | 104(6.1%) | 1# |
| Calcium Channel Blockers, no. (%) | |  | 3(13.0%) | 74(4.4%) | 0.080# |
| Nitrates, no. (%) | |  | 3(13.0%) | 42(2.5%) | 0.021# |
| Coumadin, no. (%) | |  | 2(8.7%) | 77(4.5%) | 0.285# |
| Heparin, no. (%) | |  | 0(0%) | 2(0.1%) | 1# |
| Clopidogrel, no. (%) | |  | 0(0%) | 12(0.7%) | 1# |
| Aspirin, no. (%) | |  | 1(4.3%) | 93(5.5%) | 1# |
| Statin use, no.(%) | |  | 1(4.3%) | 32(1.9%) | 0.361# |
| Diuretics, no. (%) | |  | 5(21.7%) | 145(8.5%) | 0.026 |
| Digoxin, no. (%) | |  | 4(17.4%) | 97(5.7%) | 0.042# |
| **Preoperative laboratory examination** | | | | | |
| LVEF <35%, no.(%) | |  | 0(0%) | 9(0.5%) | 1# |
| T-ch [mean(SD); mmol/L] | |  | 4.1 ± 0.9 | 4.3 ± 1.0 | 0.218 |
| BUN [mean(SD); mmol/L] | |  | 6.0 ± 2.0 | 5.9 ± 2.1 | 0.870 |
| Hb [mean(SD); g ·L^-1^] | |  | 135 ± 21 | 132 ± 19 | 0.505 |
| PLt [mean(SD); 10^3^/mm^3^] | |  | 152 ± 48 | 169 ± 58 | 0.173 |
| INR [mean(SD); seconds] | |  | 1.16 ± 0.41 | 1.09 ± 0.29 | 0.436 |
| Propensity score [median(SD)] | |  | 0.37 ±0.31 | 0.34 ±0.23 | 0.678 |
| #: Fisher’s exact test was used; ARB= angiotensin receptor blockers; ACEI= angiotensin converting enzyme inhibitors; LVEF= left ventricular ejection fraction; T-ch= serum cholesterol; BUN= serum urea nitrogen; Hb= hemoglobin; PLt= Platelet count; INR= international normalized ratio. | | | | | |

| **S3 Table L. Operative Characteristics between patients with resternotomy for postoperative bleeding or not.** | | | | | | |
| --- | --- | --- | --- | --- | --- | --- |
| Characteristics |  | Resternotomy for postoperative bleeding | | | p-value | |
|  |  | Yes (N= 23) | No (N= 1697) | |  |  |
| Redo surgery |  | 2(8.7%) | 25(1.5%) | | 0.049# | |
| Emergent operation |  | 2(8.7%) | 12(0.7%) | | 0.014# | |
| **Type of surgery, no. (%)** |  |  |  | | 0.008 | |
| Aortic valve |  | 3(13.0%) | 238(14.0%) | |  | |
| Mitral valve |  | 5(21.7%) | 363(21.4%) | |  | |
| Tricuspid valve |  | 0(0%) | 98(5.8%) | |  | |
| Complex cardiac |  | 12(52.2%) | 651(38.4%) | |  | |
| Transplant |  | 1(4.3%) | 5(0.3%) | |  | |
| Aortic |  | 2(8.7%) | 83(4.9%) | |  | |
| Others |  | 0(0%) | 259(15.3%) | |  | |
| Duration of anesthesia [mean(SD); min] |  | 353 ± 191 | 290 ± 92 | | 0.128 | |
| Duration of surgery [mean(SD); min] |  | 269 ± 140 | 246 ± 90 | | 0.458 | |
| CPB time [mean(SD); min] |  | 145 ± 68 | 122 ± 58 | | 0.057 | |
| IABA, no. (%) |  | 0(0%) | 6(0.4%) | | 1# | |
| **Intra-operative transfusion** |  |  | |  | |  |
| RBCs transfusion, no. (%) |  | 8(34.8%) | | 327(19.3%) | | 0.062 |
| FFP transfusion, no. (%) |  | 16(69.6%) | | 569(33,5%) | | < 0.001 |
| Platelet transfusion, no. (%) |  | 1(4.3%) | | 79(4.7%) | | 1# |
| **Input and Output Characteristics** |  |  | |  | |  |
| Blood loss [mean(SD); mL] |  | 778 ± 401 | | 659 ± 395 | | 0.152 |
| Urine output [mean(SD); mL] |  | 850 ± 501 | | 709 ± 417 | | 0.108 |
| Pump blood [mean(SD); mL] |  | 552 ± 112 | | 509 ± 127 | | 0.109 |
| Autologous transfusion [median(IQR); mL] |  | 425(250~650) | | 300(200~500) | | 0.160 |
| ANH [median(IQR); mL] |  | 0(0~200) | | 0(0~0) | | 0.425 |
| Introperative Crystals [mean(SD); mL] |  | 1630 ± 856 | | 1467 ± 547 | | 0.370 |
| Introperative Colloid [mean(SD); mL] |  | 783 ± 448 | | 680 ± 364 | | 0.181 |
| **Intra-operative coagulation drugs** |  |  | |  | |  |
| Prothrombin Complex Concentrate, no. (%) |  | 1(4.3%) | | 44(2.6%) | | 0.459# |
| Fibrinogen concentrate, no. (%) |  | 1(4.3%) | | 53(3.1%) | | 0.522# |
| recombinant activated factor VII, no. (%) |  | 2(8.7%) | | 4(0.2%) | | 0.002# |
| Tranexamic acid, no. (%) |  | 6(26.1%) | | 576(33.9%) | | 0.429 |
| | #: Fisher’s exact test was used; Redo surgery= ≥1 previous sternotomy; Aortic valve surgery= without ascending aortic replacement; Complex cardiac surgery= multi-valve surgery; Aortic surgery= Aortic dissections, type A and B, thoracic aortic aneurysms) or Aortic valve surgery with ascending aortic replacement; Others surgery type including ASD, VSD, LAM, ASV, CPF, PFO etal.; CPB= cardiopulmonary bypass; IABA= intra-aortic balloon pump; RBCs= red blood cells. |  | | --- | --- | | | | | | | |
