# Supplementary figures and images for: Intraoperative tranexamic acid is associated with postoperative stroke in patients undergoing cardiac surgery
Source: PLoS One. 2017 May 26;12(5):e0177011. doi: 10.1371/journal.pone.0177011 (PMC5446127; doi:10.1371/journal.pone.0177011)

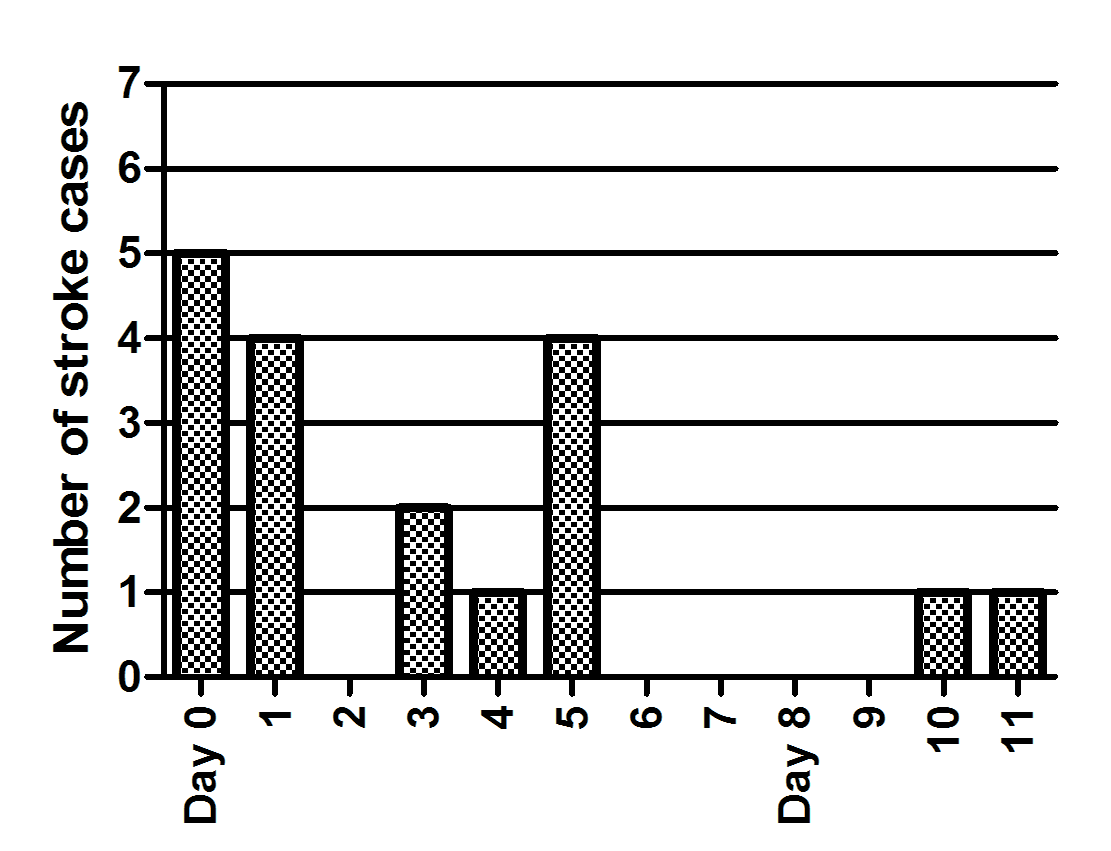

Supplement: S1 Fig — (TIF) [file pone.0177011.s004.tif]

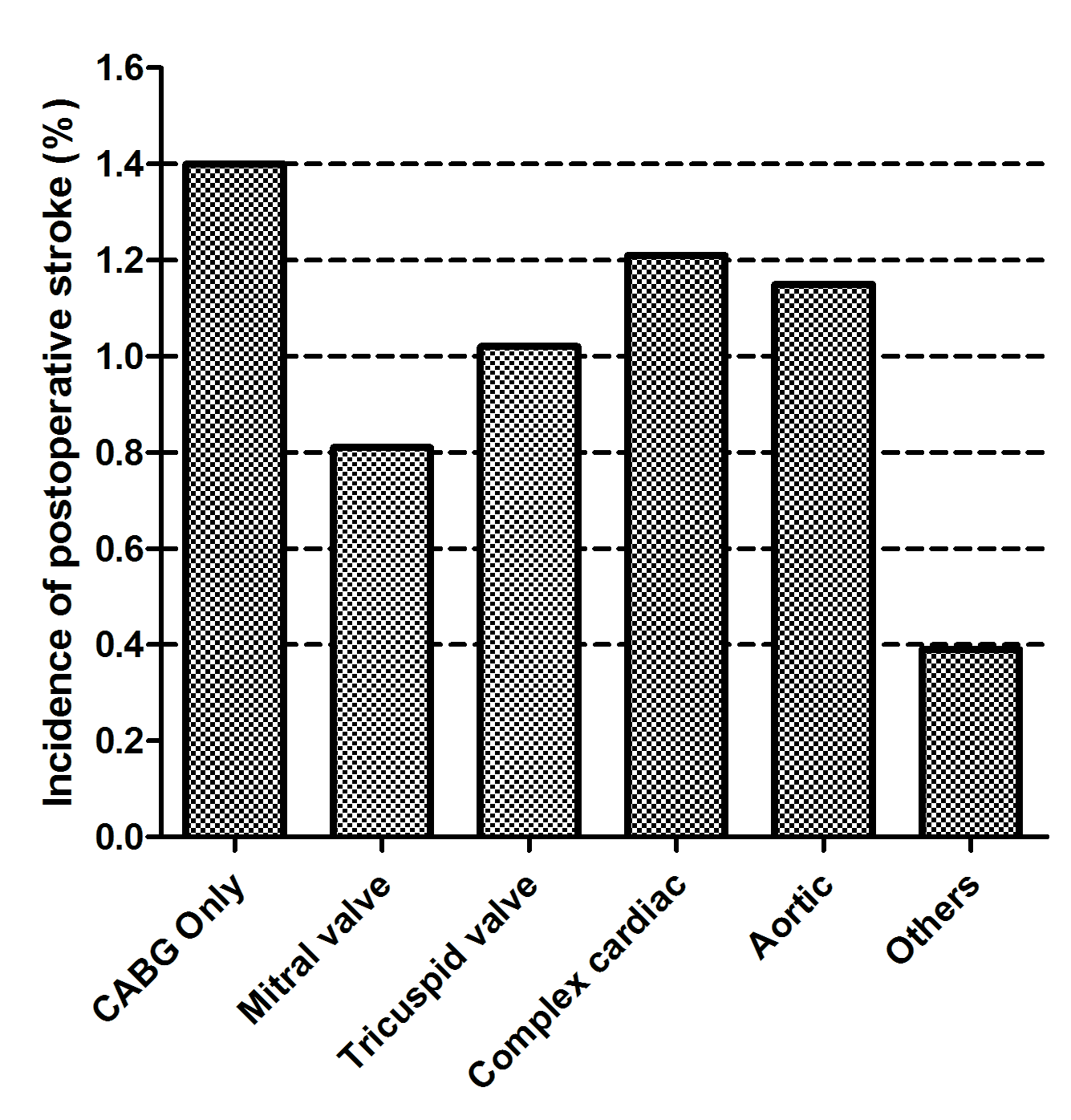

Supplement: S2 Fig — (TIF) [file pone.0177011.s005.tif]
