# Supplementary material for: Intraoperative tranexamic acid is associated with postoperative stroke in patients undergoing cardiac surgery
Source: PLoS One. 2017 May 26;12(5):e0177011. doi: 10.1371/journal.pone.0177011 (PMC5446127; doi:10.1371/journal.pone.0177011)
Supplement: S2 Text — (DOC) [file pone.0177011.s007.doc]

**Effect of** **Tranexamic Acid on Postoperative Outcomes in Patients Undergoing Cardiac Surgery-**

**A Retrospective Study Protocol**

**1. Background**

Postoperative bleeding is a major complication in cardiac surgery ^[1, 2]^. Concern about potential hemorrhagic risk of cardiac surgery may influence physicians to continue antifibrinolytic agents, thus increasing stroke risk in those patients ^[3]^. Tranexamic acid (TXA), a synthetic antifibrinolytic drug, has been increasingly used to manage patients for preventing perioperative bleeding in cardiac surgery ^[4, 5]^, acting by inhibiting tissue plasminogen and plasmin ^[6]^. Many randomized trials have found that TXA could decrease blood loss and allogeneic transfusion in patients undergoing cardiac surgery ^[3, 7, 8]^, but the security of routine administration of TXA is still uncertain ^[4, 7, 9]^. In a recent study, TXA was found occasional associated with early cerebral stroke at extubation, but not with delayed stroke ^[10]^, however, the result remains unconfirmed for lacking sufficient analysis support.

Stroke is another disaster cerebrovascular complication after cardiac surgery and is linked to a poorer outcome ^[11, 12]^. A series of risk factors have been found for postoperative stroke in previous studies, such as atherosclerosis, female gender, history of stroke and smoke, advanced age, and other comorbidities ^[13]^. Although a meta-analysis noted that either mortality or thromboembolic events was few and was not increased in TXA group compared to non-treatment group, the authors still admonish for routine use of TXA in cardiac surgery ^[14]^.

As those studies do not report complications or are underpowered, clinical safeties of TXA including the risk of adverse neurological effects such as stroke and thromboembolic events remains uncertain, as well as the mortality and morbidity ^[15]^. The aim of this study was to investigate the relationship between TXA and postoperative stroke in patients undergoing cardiac surgery.

**3. Projection and Methods**

(1) It is a retrospective study. Individual patient data of total consecutive adult patients underwent cardiac surgery with CPB was collected through medicinal record from January 1, 2010 to December 31, 2015.

(2) Inclusion criteria: From January 1, 2010, to December 31, 2015, total consecutive adult patients underwent cardiac surgery with cardiopulmonary bypass (CPB).（age≥ 18 yr）

(3) Exclusion criteria:

a) Patients with carotid artery disease, peripheral vascular disease;

b) Patients were treated with postoperative extracorporeal membrane oxygenation (ECMO).

c) Patients experienced deep hypothermic circulatory arrest

(4) Data Collection:

a) Baseline parameters: hospital number, age, sex, Height, Weight, body mass index (BMI), American Society of Anesthesiologists (ASA) classification and so on;

b) Preoperative characteristics：diagnosis, New York Heart Association(NYHA) classification, co-morbidities (hypertension, hyperlipidaemia, diabetes mellitus, chronic obstructive pulmonary disease (COPD), atrial fibrillation (AF), chronic kidney disease (CKD) or liver dysfunction, cerebrovascular disease, peripheral vascular disease, myocardial infarction (MI), infective endocarditic, shock, anemia and so on), preoperative medication, history of smoking, requiring for intra-aortic balloon pump(IABP), laboratory results before operation within one week, and so on.

c) Intraoperative characteristics: redo and emergent surgery, surgery type, deep hpothermic circulatory arrest, duration of cardiopulmonary bypass (CPB), anesthesia and operation time, requiring for intra-aortic balloon pump(IABP), blood loss, urine output, pump blood, acute normovolemic hemodilution, cell salvage transfusion, introperative crystals and colloid, blood transfusions, coagulation drugs and arterial blood gas analysis, intra-IABA use, and so on.

d) Postoperative characteristics: mortality, cerebrovascular adverse events (stroke, delirium and so on), postoperative pulmonary infection and other infection, delayed healing of wound, resternotomy for postoperative bleeding and the reason, ventilation time and length of hospital stay mortality, new-onset atrial fibrillation, septicopyemia, need for continuous renal replacement therapy, reoperation for delayed healing of wound, blood transfusions, laboratory characteristics, incidence of 6-month readmission and the reason, and so on.

(5) Outcome：

Main end points：Postoperative Stroke. Postoperative stroke was identified as a new onset of neurological deficit symptom following an ischemic cerebrovascular accident and confirmed by radiological data (computed tomography or magnetic resonance imaging), which lasting more than 24 hours ^[10, 11]^

Secondary end points：

a) clinical end points during hospital stay: mortality, cerebrovascular adverse events (Stroke, Delirium), postoperative pulmonary infection and other infection, delayed healing of wound, resternotomy for postoperative bleeding and the reason, new-onset atrial fibrillation, septicopyemia, need for continuous renal replacement therapy, reoperation for delayed healing of wound, ventilation time and length of hospital stay, incidence of 6-month readmission and the reason, and so on.

b) blood loss and transfusions: blood loss, perioperative transfusions, resternotomy for postoperative bleeding.

(6) The student’s t-test was used to test for normal continuous variables, Mann–Whitney U-test was used for non-normality continuous variables, and the Chi-squared or Fisher’s exact tests were used for categorical data. Multivariate regression analyses were used to reduce distortion by confounding variables. *p* <0.05 was considered to be statistically significant and all reported *p* values were 2-sided.

(7) The principal investigators were registered doctors in our institution and graduate students.

(8) Study flowchart

**
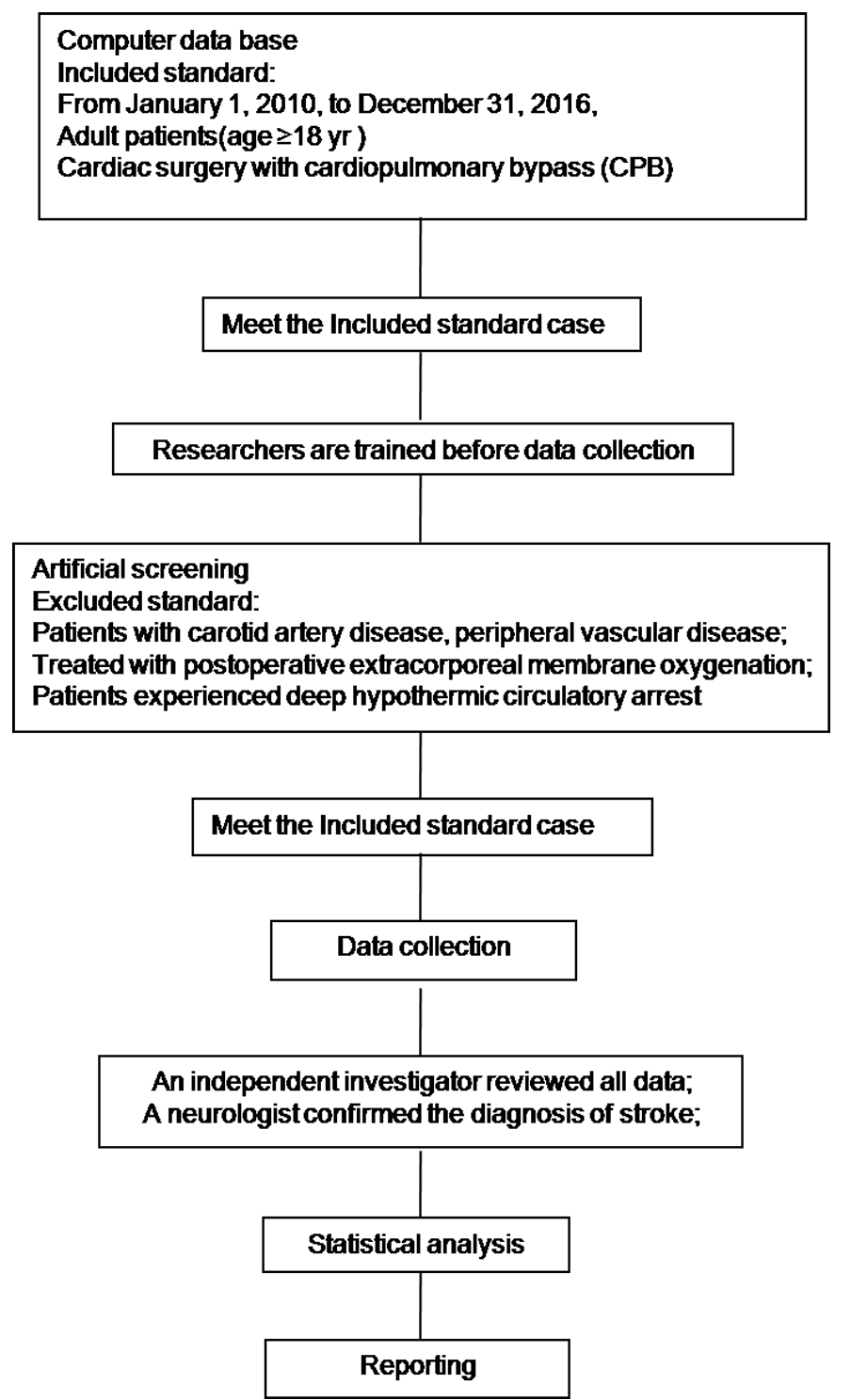
**

**4. Sample size calculation**

The incidence rate of postoperative stroke was 0.031, administration of TXA was found associated with early stroke (OR =2.03) in a previous cohort ^[10]^. Regression for a dichotomous predictor of TXA on postoperative stroke was performed and we estimated that 658 patients were required to provide 80% power to detect a two sided difference, with a type I error probability of 0.05. Assuming that follow-up lost rate was 10 %, then a total of 1086 cases are needed. Analysis was computed using G-Power (version 3.1; Informer Technologies, Inc.).

**5. References**

[1] Murphy GJ, Reeves BC, Rogers CA, Rizvi SI, Culliford L, Angelini GD. Increased mortality, postoperative morbidity, and cost after red blood cell transfusion in patients having cardiac surgery. Circulation. 2007; 116(22):2544-52. Epub 2007 Nov 12.

[2] Mehta RH, Sheng S, O'Brien SM, Grover FL, Gammie JS, Ferguson TB, Peterson ED; Society of Thoracic Surgeons National Cardiac Surgery Database Investigators. Reoperation for bleeding in patients undergoing coronary artery bypass surgery: incidence, risk factors, time trends, and outcomes. Circ Cardiovasc Qual Outcomes. 2009; 2(6):583-90. doi: 10.1161/CIRCOUTCOMES.109.858811. Epub 2009 Oct 6.

[3] Henry DA, Carless PA, Moxey AJ, O'Connell D, Stokes BJ, Fergusson DA, Ker K. Anti-fibrinolytic use for minimising perioperative allogeneic blood transfusion. Cochrane Database Syst Rev. 2011; (3):CD001886. doi: 10.1002/14651858.CD001886.pub4.

[4] Ker K, Edwards P, Perel P, Shakur H, Roberts I. Effect of tranexamic acid on surgical bleeding: systematic review and cumulative meta-analysis. BMJ. 2012 ; 344:e3054. doi: 10.1136/bmj.e3054.

[5] Dell'Amore A, Caroli G, Nizar A, Cassanelli N, Luciano G, Greco D, Dolci G, Bini A, Stella F. Can topical application of tranexamic acid reduce blood loss in thoracic surgery? A prospective randomised double blind investigation. Heart Lung Circ. 2012; 21(11):706-10. doi: 10.1016/j.hlc.2012.06.016. Epub 2012 Jul 28.

[6] Sigaut S, Tremey B, Ouattara A, Couturier R, Taberlet C, Grassin-Delyle S, Dreyfus JF, Schlumberger S, Fischler M. Comparison of two doses of tranexamic acid in adults undergoing cardiac surgery with cardiopulmonary bypass. Anesthesiology. 2014; 120(3):590-600. doi: 10.1097/ALN.0b013e3182a443e8.

[7] Ortmann E, Besser MW, Klein AA. Antifibrinolytic agents in current anaesthetic practice. Br J Anaesth. 2013; 111(4):549-63. doi: 10.1093/bja/aet154. Epub 2013 May 9.

[8] Wang G, Xie G, Jiang T, Wang Y, Wang W, Ji H, Liu M, Chen L, Li L.. Tranexamic acid reduces blood loss after off-pump coronary surgery: a prospective, randomized, double-blind, placebo-controlled study. Anesth Analg. 2012; 115(2):239-43. doi: 10.1213/ANE.0b013e3182264a11. Epub 2011 Jul 7.

[9] Ker K, Prieto-Merino D, Roberts I. Systematic review, meta-analysis and meta-regression of the effect of tranexamic acid on surgical blood loss. Br J Surg. 2013; 100(10):1271-9. doi: 10.1002/bjs.9193. Epub 2013 Jul 9.

[10] Hedberg M, Engstrom KG. Stroke after cardiac surgery--hemispheric distribution and survival. Scand Cardiovasc J. 2013; 47(3):136-44. doi: 10.3109/14017431.2012.737016. Epub 2012 Nov 1.

[11] Tarakji KG, Sabik JF 3rd, Bhudia SK, Batizy LH, Blackstone EH. Temporal onset, risk factors, and outcomes associated with stroke after coronary artery bypass grafting. JAMA. 2011; 305(4):381-90. doi: 10.1001/jama.2011.37.

[12] LaPar DJ, Quader M, Rich JB, Kron IL, Crosby IK, Kern JA, Tribble CG, Speir AM, Ailawadi G. Institutional Variation in Mortality After Stroke After Cardiac Surgery: An Opportunity for Improvement. Ann Thorac Surg. 2015; 100(4):1276-82; discussion 1282-3. doi: 10.1016/j.athoracsur.2015.04.038. Epub 2015 Jul 16.

[13] Mérie C, Køber L, Olsen PS, Andersson C, Jensen JS, Torp-Pedersen C. Risk of stroke after coronary artery bypass grafting: effect of age and comorbidities. Stroke. 2012; 43(1):38-43. doi: 10.1161/STROKEAHA.111.620880. Epub 2011 Oct 27.

[14] Ngaage DL, Bland JM. Lessons from aprotinin: is the routine use and inconsistent dosing of tranexamic acid prudent? Meta-analysis of randomised and large matched observational studies. Eur J Cardiothorac Surg. 2010; 37(6):1375-83. doi: 10.1016/j.ejcts.2009.11.055. Epub 2010 Feb 1.

[15] Martin J, Cheng D. Tranexamic acid for routine use in off-pump coronary artery bypass surgery: evidence base "fait accompli" or more research needed. Anesth Analg. 2012; 115(2):227-30. doi: 10.1213/ANE.0b013e31825b6746.

**Study protocol** **in original language**

**氨甲环酸应用对心脏手术患者术后结局**

**影响的回顾性研究-研究方案**

**一、研究背景**

心脏手术体外循环导致的出血是术后最主要的并发症之一。对心脏术后出血风险的关注可能影响医生连续使用抗纤维蛋白溶解药，因而可能增加这些患者术后脑梗塞的风险。一种合成的抗纤维蛋白溶解药氨甲环酸已被用来预防心脏术后出血，氨甲环酸通过与纤溶酶和纤溶酶原上的纤维蛋白亲和部位的赖氨酸结合部位(LBS)强烈吸附,阻抑了纤溶酶、纤溶酶原与纤维蛋白结合,从而强烈地抑制了由纤溶酶所致纤维蛋白分解。很多随机、对照研究已经证实氨甲环酸能减少心脏手术患者术中出血和围术期异体血制品输注。最近一项回顾性研究偶然发现氨甲环酸与气管导管气管时的早期脑梗塞有关，然而因缺乏足够的统计分析支持而使结果变得不确定。

术后脑梗是心脏手术后另一项严重的脑血管并发症，而且可使死亡率增加3-6倍。最近一项Meta分析指出心脏手术后死亡和栓塞事件发生率低，与未使用抗纤维蛋白溶解药相比，虽然心脏手术中使用氨甲环酸并不增加术后死亡率和栓塞事件的发生，但作者还是质疑心脏手术中常规使用氨甲环酸的必要性。

氨甲环酸使用的临床安全性还未得到证实，包括脑梗塞和其它栓塞事件等并发症。本研究旨在回顾性分析术中输注氨甲环酸对CPB下心脏手术术后脑梗塞的影响。

**二、研究目的：**

分析心脏手术中氨甲环酸对术后脑梗塞的影响。

**三、研究设计和方法：**

（1）回顾性研究。通过查阅病历，收集浙江大学医学院附属第二医院2010年1月1日-2015年12月31日体外循环下心脏手术患者临床资料。

（2）入选标准： 成人患者行体外循环下心脏手术（年龄：≥18岁）

（3）排除标准：

①术前存在颈动脉或外周血管疾病；

②术后使用体外膜肺氧合(extracorporeal membrane oxygenation，ECMO)；

③术中采用深低温停循环技术。

（4）数据收集：

①基本资料：年龄，性别，体重，身高，BMI, ASA分级，血型等等

②术前情况：术前诊断，心脏NYHA分级，基础疾病（高血压、糖尿病、肝脏疾病、肾脏疾病、血液系统疾病、心脑血管疾病、呼吸系统疾病、外周血管疾病、心内膜炎等等），术前疾病诊治过程，个人生活史（吸烟、饮酒史等等），术前用药，术前一周内血液、尿液等化验结果，影像学检查，术前有无休克、心衰及是否使用球囊反搏等等

③术中情况：既往开胸手术史，深低温下体外循环、急诊、手术类型、出血量、尿量、机血、术中异体血种类和量、血液稀释和自体血回收量，晶胶液体量，止血药物种类和量，手术时间，麻醉时间、体外循环时间，体温，术中主动脉球囊阻断（IABP）使用，血气分析结果，术中检查化验，术中凝血用药等等。

④术后情况：死亡率，二次手术率及原因，术后出血和出入量，切口愈合延迟，术后异体血种类和量，晶胶液体量， 机械通气时间， ICU停留天数，总住院时间，术后并发症：脑血管不良事件（脑卒中、脑出血、昏迷、谵妄）、肾功能衰竭（连续肾脏替代治疗）、脓毒血症、肺部感染、其它感染及部位等，术后检查化验结果，出院后6个月再入住院及原因等等。

（5）观察指标：

主要指标：术后30天内脑梗塞发生率。术后脑梗塞定义为术后因脑缺血意外新发神经系统症状持续达24小时及以上，并有影像学（CT或MRI）证据。

次要指标：

①住院期间临床结局指标：需要血液透析，死亡率和其它并发症，机械通气时间，ICU和住院时间。

②出血和输血：术中出血量、围术期异体血制品输注量、因出血二次手术率；

（6）统计学方法：计量资料用均数±标准差，非正态计量资料分布用中位数和四分位数，计数资料用数值或百分比表示。计数资料采用 *X*^2^检验或Fish确切概率法，计量资料采用t检验或U检验，非正态分布资料采用非参数检验方法。采用多因素变量回归分析，双侧*p*< 0.05为差异有统计学意义。

（7）主要调查人员为获得授权的本院医生和研究生。

（8）实验操作流程图

电脑软件查询

纳入查询：

2010年1月1日-2015年12月31日

体外循环下心脏手术

成人手术（年龄≥18岁）

数据收集前培训

数据核对检查

数据收集

统计分析

总结报告

符合病例

人工筛选：

排除标准：

术前存在颈动脉或外周血管疾病；

术后使用体外膜肺氧合

术中采用深低温停循环技术

符合病例

**四、样本量计算**

有研究报道体外循环心脏术后住院期间脑梗塞发生率为3.1%，氨甲环酸与术后早期脑梗塞的优势比（OR值）是2.03。主要效能分析旨在显示氨甲环酸增加术后30天内脑梗塞发生，采用Logistic回归分析检验，双侧α= 0.05，检测效能β为80%。共658例，每组需要329例。根据主要观察指标，我们使用G-power3.1.9.2软件计算样本大小。假设失访率为10%，每组需要362例，总共1086例。
