# Supplementary material for: Intraoperative tranexamic acid is associated with postoperative stroke in patients undergoing cardiac surgery
Source: PLoS One. 2017 May 26;12(5):e0177011. doi: 10.1371/journal.pone.0177011 (PMC5446127; doi:10.1371/journal.pone.0177011)
Supplement: S3 Text — (PDF) [file pone.0177011.s008.pdf]

Approval of the Ethics Committee of Second Affiliated Hospital of Zhejiang University

Accepted Number：Study 2016-018	Approval number: (2016 yr) Ethical Study (021)				
Project Name	Tranexamic Acid on Postoperative Outcomes in Patients Undergoing Cardiac Surgery				
Application Type	□Drug/Device Clinical Trials ☒Clinical Research □New Technology □Post-Market Products				
Drug/Device Type	/	Specification: /	Test Category	/	
Sponsor	The Second Affiliated Hospital of Zhejiang University				
CRO	/				
Undertake Department	Anesthesiology	PI	Min YAN		
☒ In Charge □ Participate	Leader Institute	/			
Review documents	Initial Examination Document(Quick Review Results in April 19, 2016: Approve);<br>Ethics Review Application Form, Research Program (V1.0, March 29, 2016), Informed Consent (V1.0, March 29, 2016), Case Report Form (V1.0, March 29, 2016), Proof of Ethical Training, Resume of Principal Investigator and Participants, Commitment of Research, Responsibilities Table				
Review Form	□ Conference Review	☒ Quick Review			
Date of Review: April 19, 2016	Conference Place: /				
Review Committee	Rong-zhi XUI, Hong YU				
Conclusions	1. The body ethics review committee review; It is approved to follow the latest program after the Ethics Review Committee review the material.<br>2. The study process will be subject to under continuous review by the Ethics Review Committee Board, with a frequency since the date of approval: □ Six month ☒Twelve month □Other____<br>3. This Ethics Review Committee Board is empowered to change the frequency of continuous review according to actual progress.				
Approve<br>Signature of Director / Deputy Director: Date: April 19, 2016<br>The Ethics Committee of Second Affiliated Hospital of Zhejiang University					
Remarks	1. The study shall be implemented within one year since the date of approval. If the application is overdue, the approval shall be abolished.<br>2. It should be compliance with the approval strictly. Any changes of the program should be approved by the Ethics Committee after submitting an application.<br>3. SAE Report is required and we have the right to make new decisions according to the SAE Report<br>4. It should be submitted for continuous review one month in advance.<br>5. Any program violations and early termination should be provided with a written report.<br>6. Provided a summary report when the study was completed.<br>7. Provided a written report of the Ethics important decision timely.				

浙江大学医学院附属第二医院人体研究伦理委员会伦理批件

受理编号：研 2016-018　　批件号：（2016 年度）伦审研第（021）号

项目名称	围术期氨甲环酸应用对心脏手术后临床结局的影响				
申请类型	□ 药/械临床试验　☒ 临床科研　□ 医疗新技术　□上市后产品研究				
药/械类别	/	规格：	/	试验类别	/
申办方	浙江大学医学院附属第二医院				
CRO	/				
承担科室	麻醉科	主要研究者 PI	严敏		
☒负责　□参加	组长单位	/			
审查文件	初始审查文件（2016 年 4 月 19 日快速审查结果：同意）<br>伦理审查申请表、研究方案（V1.0，2016.03.29）、知情同意书（V1.0，2016.03.29）、病例报告表（V1.0，2016.03.29）、伦理培训证明、主要研究者简历及参加研究人员简介、研究者承诺书、岗位职责表				
审查形式	口会议审查　☒快速审查				
审查日期	2016. 4. 19	会议地点：/			
审查委员	徐荣臻、余红				
审查结论	1. 经本人体伦理审查委员会审查，同意按照最新方案执行。<br>2. 该研究进行过程中将接受本人体研究伦理审查委员会的持续审查，持续审查频率为研究批准之日起：口6个月　☒12个月　口其它______<br>3. 本人体研究伦理审查委员会有权根据实际进展情况改变持续审查频率。				
同意<br>主任/副主任委员签名：[签名]　日期：2016 年 4 月 19 日<br>浙江大学医学院附属第二医院人体研究伦理委员会（盖章）					
备注	1. 研究应在批准之日起 1 年内实施，逾期未实施，本批件自行废止。<br>2. 严格遵从批准的方案实施，研究过程中对方案、知情同意书等材料的任何修改，请提交修改申请，得到伦理委员会批准后方可继续实施。<br>3. 按要求书面上报 SAE，本伦理委员会有权根据 SAE 报告作出新的决定。<br>4. 自同意研究之日起，根据审查频率提前 1 个月提交持续审查申请。<br>5. 方案违背、提前终止均应提供书面报告。<br>6. 研究结束提供总结报告。<br>7. 及时书面报告中心伦理的重要决定。				
